# Intestinal alkaline phosphatase (IAP, IAP Enhancer) attenuates intestinal inflammation and alleviates insulin resistance

**DOI:** 10.3389/fimmu.2022.927272

**Published:** 2022-07-25

**Authors:** Chenzhe Gao, Marwa Yagoub Farag Koko, Mingxing Ding, Weichen Hong, Jianping Li, Na Dong, Mizhou Hui

**Affiliations:** ^1^ The Laboratory of Molecular Nutrition and Immunity, Institute of Animal Nutrition, Northeast Agricultural University, Harbin, China; ^2^ College of Food, Northeast Agricultural University, Harbin, China; ^3^ Changchun Jiahe Surgery Hospital, Changchun, China

**Keywords:** intestinal inflammation, intestinal alkaline phosphatase, lipopolysaccharide, adenosine, dephosphorylation, signalling pathway

## Abstract

In this study, we investigated the effects of intestinal alkaline phosphatase (IAP) in controlled intestinal inflammation and alleviated associated insulin resistance (IR). We also explored the possible underlying molecular mechanisms, showed the preventive effect of IAP on IR *in vivo*, and verified the dephosphorylation of IAP for the inhibition of intestinal inflammation *in vitro*. Furthermore, we examined the preventive role of IAP in IR induced by a high-fat diet in mice. We found that an IAP + IAP enhancer significantly ameliorated blood glucose, insulin, low-density lipoprotein, gut barrier function, inflammatory markers, and lipopolysaccharide (LPS) in serum. IAP could dephosphorylate LPS and nucleoside triphosphate in a pH-dependent manner *in vitro*. Firstly, LPS is inactivated by IAP and IAP reduces LPS-induced inflammation. Secondly, adenosine, a dephosphorylated product of adenosine triphosphate, elicited anti-inflammatory effects by binding to the A_2A_ receptor, which inhibits NF-κB, TNF, and PI3K-Akt signalling pathways. Hence, IAP can be used as a natural anti-inflammatory agent to reduce intestinal inflammation-induced IR.

## Introduction

Type 2 diabetes (T2DM) is a major global health problem, and its incidence increases every year. In 2019, the latest epidemiological data showed that the number of adults with T2DM reached 463 million (1). A hallmark of T2DM is insulin resistance (IR), which is associated with metabolic disorders (2). Despite the rapid development of global technology, no complete consensus on the pathogenesis of IR has been presented, illustrating the complexity and refractory nature of IR.

Intestinal inflammation is one of the most important causes of IR (3). In mammals, the gut is the largest immune organ that contains secondary immune organs, the structure of the tertiary lymphoid, and immune cells scattered throughout the lamina propria and intraepithelial spaces of the intestine, which work together to protect the body from foreign substances (4). Moreover, inflammatory mediators such as IL-13, TNF-α, and IFN-γ damage the integrity of the epithelial barrier and increase the degree of intestinal inflammation (5). Therefore, strategies that can reduce intestinal inflammation must be developed.

LPS acts as a toxin by overstimulating the innate immune signalling of Toll-like receptor 4 (TLR4), which induces an exacerbated inflammatory response (6). Nevertheless, IAP can detoxify bacterial LPS *via* dephosphorylation (7). Lipid A is responsible for LPS toxicity and composed of two phosphate groups coupled to glucosamines, allowing LPS to bind to TLR4, triggering an inflammatory response (7). However, the removal of one of the phosphate groups from lipid A by IAP produces a monophosphoryl lipid A, which is 100 times less toxic than unmodified lipid A (8).

In the initiation of inflammation, extracellular purines (adenosine, ADP, and ATP) and pyrimidines (UDP and UTP), which are released from host cells including nerve termini, immune cells, injured or dead cells, and the commensal bacteria that reside in the gut lumen, stimulate purinergic receptors in autocrine and paracrine ways (9). Once released, extracellular ATP (eATP) is rapidly hydrolysed to ADP, AMP, and adenosine by IAP (10). The latter exerts intestinal protective and anti-inflammatory effects by binding to one of the four adenosine receptors A_1_, A_2A_, A_2B_, and A_3_ (11). M. Estaki et al. (6) reported that metabolic syndrome develops in IAP knockout mice fed with high fat, but this condition can be alleviated by oral IAP. In addition, IAP overexpression attenuates the attenuated development of WD-induced atherosclerosis by limiting the translocation of gut-derived LPS or reducing plasma lipids (12). Therefore, oral IAP supplementation represents a novel therapy to counteract the chronic inflammatory state leading to frailty and age-related diseases in humans (13).

IAP is a phosphomonoesterase that catalyses the hydrolysis of non-specific phosphate ester bonds, whose activity is positively correlated with pH (14). We aimed to build on these findings by determining whether pharmacologic approaches that trigger IAP activity by increasing pH in the gut could reduce intestinal inflammation and elicit a palliative effect on IR. Earlier laboratory studies suggested the use of oral hydrotalcite as an IAP enhancer, which can improve IAP activity by regulating intestinal pH to alkalinisation in the treatment of LPS-associated intestinal inflammatory diseases (15).

However, studies have yet to explore how IAP deficiency is linked to these diseases. Therefore, this study was designed to investigate the protective role of IAP during intestinal inflammation and reveal the underlying molecular mechanisms of action that would delay the further development of inflammatory bowel disease and IR. This study also aimed to explore the safety and initial efficacy of IAP and provide a basis for using IAP as a potential biological treatment for IR caused by intestinal inflammation.

## Materials and methods

### Simulation of gastrointestinal digestive properties by an IAP enhancer *in vitro*

Simulated gastric juice was prepared by supplementing 2 g NaCl, 1.12 g KCl, 0.11 g CaCl, and 0.4 g KH_2_PO_4_, and its volume was fixed to 1,000 ml in distilled water. Then, the juice was sterilised (121°C, 15 min) and added to 3 g/l pepsin (Sigma, USA). Buffer solution broth was prepared by adjusting the pH to 2.5 with 1 mol/l HCl. Simulated intestinal juice was prepared by supplementing 10 g/l trypsin (Sigma, USA) and 3.0 g/l bile salt (Sigma, USA) added into an artificial gastric fluid, and pH was adjusted to 6.5 with 1 mol/l NaHCO_3_.

Hydrotalcite, oat flour, and IAP enhancer (composed of oatmeal, shell powder, aluminium hydroxide, calcium carbonate, magnesium carbonate, xylitol, etc.) were used as raw materials (through the puffing extrusion method, Qingdao Dr. Hui Biological Technology Co., Ltd., China, SC122370684001877; **Table S1**) by 10% (W/V) access to artificial gastric juice and digested in a shaker at 100 r/min and 37°C for 1 h. The artificial intestinal juice was added at 10% (W/V) and digested in a shaker at 100 r/min and 37°C for 6.5 h, i.e., simulated duodenal digestion for 0.5 h and intestinal digestion and absorption for 6 h (16).

### IAP and LPS activity

Tris–HCl buffer (50 mmol/l) was adjusted to pH 5.0, 5.5, 6.0, 6.5, 7.0, 7.5, and 8.0 by using HCl (1 mol/l). Then, 0.1 ml IAP (IAP [molecular weight of 59 kDa and purity of 95.71%] purified from fresh pig intestinal mucosa *via* ion chromatography; College of Food, Northeast Agricultural University, China) was added to 0.9 ml buffer solution with different pH levels. The IAP activity was measured using an alkaline phosphatase detection kit (TE0005, Nanjing Jiancheng Biotechnology Co., Ltd., China) (17).

IAP (0.5 mL, 400 U/ml) was added to Tris–HCl buffer (4.5 ml, 50 mmol/l), and the final pH was adjusted to 5.0, 5.5, 6.0, 6.5, 7.0, 7.5, and 8.0. LPS (*E. coli* 0111: B4, Sigma, USA) was diluted to 0.1 mg/ml with Tris–HCl buffer (50 mmol/l, pH = 8.0). Then, 10 μl of the diluted LPS solution (pH = 8.0) was added to 990 µl of IAP solutions at pH 5.0–8.0. and incubated at 37°C for 180 min. IAP was diluted to 1, 5, 10, 20, and 40 U/ml by using Tris–HCl buffer (50 mmol/l, pH = 8.0). Afterwards, 10 µl of LPS (0.1 mg/ml) was added to 990 µl of IAP solution with different IAP activities to obtain an LPS concentration of 1 µg/ml. The mixtures were incubated at 37°C for 180 min. LPS activity was determined using a quantitative Limulus endotoxin detection kit (EC32545S, Xiamen Biotechnology Co., Ltd., China) *via* the chromogenic matrix method (18).

### Free phosphate

In this procedure, 950 µl of IAP at 40.00 U/ml (pH 8.0) and 0.40 U/ml (pH 8.0) at different pH levels was used in this experiment. Then, 50 µl of a solution containing ATP, adenosine diphosphate (ADP), adenosine monophosphate (AMP), uridine triphosphate (UTP), uridine diphosphate (UDP), uridine monophosphate (UMP), guanosine triphosphate (GTP), guanosine diphosphate (GDP), guanosine monophosphate (GMP), cytidine triphosphate (CTP), cytidine diphosphate (CDP), cytidine monophosphate (CMP), thymidine triphosphate (TTP), thymidine diphosphate (TDP), thymidine monophosphate (TMP) (10 mmol/l, YuanYe Biotechnology Co., Ltd., China) with the same pH was added. The mixture was incubated at 37°C for 60 min. Afterwards, 10 µmol/l CD39 and 10 µmol/l CD73 (Sigma, USA) were prepared with Tris–HCl buffer (50 mmol/l, pH = 7.4) and mixed at equal volumes. Subsequently, 950 µl of CD39 and CD73 mixture was added, and 50 µl of ATP, ADP, and AMP solutions was added. The solution was incubated at 37°C for 60 min. Free phosphate was assessed using a phosphate assay kit (C006-1-1, Nanjing Jiancheng Biotechnology Co., Ltd., China) in accordance with the manufacturer’s protocol.

### Animal experiment

Twelve-week-old male C57BL/6J mice (Long-Life Biotechnology, Liaoning, China) were maintained in filter-top cages in a temperature-controlled room (22°C–24°C) with a 12-h light/12-h dark diurnal cycle and given food and water ad libitum in accordance with the regulations designated and approved by the Northeast Agricultural University Committee on Animal Resources (UCAR), which adheres to FDA and NIH animal care guidelines and reviews all animal protocols prior to approval (NEAUEC20220231).

The mice were placed on a high-fat diet (HFD: D12451; 35 E% from carbohydrate, 20 E% from protein, and 45 E% from fat, Research Diets). After 14 weeks of HFD treatment, those that exhibited obesity and IR, as measured by fasting blood glucose ≥11.1 mmol/l, were used in the study (n = 32). HFD-fed mice were randomly divided into four groups. 1) In the control group, mice (n = 8) were administered with a control vehicle; 2) in the IAP group, mice (n = 8) were given 100 U/100 g/day IAP; 3) in the IAP-enhancer group, mice (n = 8) were provided with 5 g/1,000 g/day IAP enhancer; and 4) in the IAP + IAP enhancer group, mice (n = 8) were treated with 100 U/100 g/day IAP + 5 g/1,000 g/day IAP enhancer. The treatments were administered by gavage at 9 a.m. for 4 weeks (19–21). As a low-fat diet (LFD) control group (n = 8), the mice were placed on LFD (D12450HL; 70 E% from carbohydrate, 20 E% from protein, and 10 E% from fat, Research Diets).

### Basic indicators

Body weight was measured using a scale (LE/KA, China). Faecal samples were collected at baseline and 4 weeks after treatments. Urine was collected *via* bladder massage at 3 p.m. (6 h after the experiment started) and after 24 h to measure pH. Urine samples were collected by gentle abdominal pressure and analysed with a BW-901 urine analyser (Mindray, China). Blood samples were drawn from the tip of the incised tail after the mice fasted for 8 h, and analysis was completed within 24 h. GTT was performed after overnight (8:00 p.m.–8:00 a.m.) fasting. The glucose levels of the tail vein blood samples were measured 0, 15, 30, 60, and 120 min after oral administration of glucose (2 g/1,000 g for mice). Fasting and postprandial blood glucose, triglyceride, cholesterol, low-density lipoprotein, and high-density lipoprotein were measured using a BS-280 automatic biochemical analyser (Mindray, China). Fasting and postprandial insulin levels were determined *via* radioimmunoassay by using UniCel DxI800 automatic chemiluminescence immunoassay (Beckman, USA).

### Faecal IAP activity

In this procedure, 0.1 g of fresh faecal sample was taken from the mice; each sample was added with 5 ml of Tris–HCl buffer (50 mmol/l, pH = 8.0), mixed, and centrifuged at 12,000 r/min and 4°C for 10 min. The supernatant was diluted to 100-fold, and faecal IAP levels were determined using an alkaline phosphatase detection kit (TE0005, Nanjing Jiancheng Biotechnology Co., Ltd., China). The result was reported as units per gram (22).

### Plasma LPS, CRP, GLP-1, TNF-α, IL-6, IL-17, IFNγ, and IL-22

Plasma was collected from the LFD control group. Then, the plasma was added to a processing solution (Xiamen Limulus Reagent Biotechnology Co., Ltd., China) at a ratio of 1:9, and the mixture was incubated in a water bath at 70°C for 10 min and cooled for 3 min. LPS at final concentrations of 0.00, 2.50, 6.25, and 12.50 pg/ml was used to generate a standard curve for plasma LPS measurements. The plasma samples to be tested were added to the same processing solution at a ratio of 1:9, and the mixture was incubated in a water bath at 70°C for 10 min and then cooled for 3 min. Plasma LPS was measured using a Limulus endotoxin detection kit (EC32545S, Xiamen Biotechnology Co., Ltd., China). Every obtained result was multiplied by 10-fold because of sample dilution (20).

The collected plasma and the levels of CRP, GLP-1, TNF-α, IL-6, IL-17, IFNγ, and IL-22 were measured using ELISA kits (Mlbio, China).

### Intestinal permeability assay

Six-centimetre segments of the intestine were removed, rinsed with ice-cold saline, everted, filled with 700 ml of PBS, and ligated at both ends. The filled jejunum segments were incubated in PBS containing 1 mol/l FITC-dextran (70 kDa; Sigma) or 0.4 mg/ml HRP (type IV, 300 units/mg solid, Sigma-Aldrich Co.). The jejunum sacs were removed after 45 min, and the contents of each sac were collected carefully using a 1-ml syringe. The amount of FITC-dextran transversing the jejunum was quantified by using a fluorescence plate reader at 521 nm. The HRP activity in the contents of each sac was determined spectrophotometrically based on the rate of pyrogallol oxidation (23).

Evans blue penetration assay: Briefly, Evans blue dye (4% in saline) was administered at 4 ml/kg *via* the tail vein for 2 h. After being anesthetised with 1% pentobarbital, the mice were perfused with normal saline to wash residual dye out of the blood vessels. Intestinal samples were then collected to evaluate intestinal barrier disruption. Evans blue was extracted from tissue homogenates incubated in formamide at 60°C for 24 h. Then, the infiltrated Evans blue was quantified using a spectrophotometer at 620 nm. The content of the dye was calculated with a standard curve method (24).

### Histological staining

The collected jejunum tissues were fixed with 4% paraformaldehyde and embedded in paraffin. Paraffin-embedded jejunum tissues were sectioned (4 μm in thickness), quickly stained with haematoxylin and eosin (HE), mounted on slides, and examined using a US Moticam 3000 photomicrography imaging system for observations and photography.

### Cell culture

All the cell lines passed the mycoplasma contamination detection. HT-29 cells (Hubei Yuze Pharmaceutical Technology Co., Ltd., China), which were used as an *in vitro* model to study absorption, transport, and secretion by intestinal cells, were inoculated in McCoy’s 5A medium (HyClone, USA) containing 1% penicillin–streptomycin dual antibodies (Solarbio, China) and 10% FBS (HyClone, USA) in T-75 culture bottles. They were cultured in a humidified 5% CO_2_ incubator at 37°C. On the experimental day, the cells were incubated in RPMI-1640 medium (HyClone, USA) containing 10% FBS. Then, cells with a density of 2.0 × 10^4^ cells/well were added to a well plate and cultured at 37°C for 24 h. In accordance with the manufacturer’s protocol, human leucocytes from healthy participants were freshly prepared using a human peripheral blood leucocyte separation solution kit (P8670, Solarbio, China). Freshly extracted human leucocytes were resuspended in RPMI-1640 medium supplemented with 10% FBS. They were added onto the top of a confluent HT-29 cell monolayer at a density of 1.0 × 10^5^ cells/well. Physical examinations, electrocardiography, and routine laboratory studies on all healthy participants before the start of the experiment showed normal results. The healthy participants were not taking any prescription medications and tested negative for hepatitis B surface antigen and human immunodeficiency virus infection. This study was reviewed and approved by the Institutional Changchun Jiahe Surgical Hospital (Changchun, Jilin Province; Ethical Approval No. 2019712) and complied with the Declaration of Helsinki including current revisions and the Good Clinical Practice guidelines. Written informed consent was obtained from all study participants.

An LPS-induced intestinal inflammation model of HT-29 cells + freshly extracted human leucocytes was established by adding 100 μl of LPS to each well, and the final concentrations were 0.10, 0.20, 1.00, and 2.00 ng/ml. Then, 100 μl of IAP (40 U/ml), 100 μl of LPS (1.0 ng/ml), 50 μl of IAP (4, 20, 40, 80 U/ml) + 50 μl of LPS (2.0 ng/ml), 50 μl of IAP (80 U/ml), and 50 μl of LPS (2.0 ng/ml) were incubated for 3 h in advance or at the same time; IAP (80 U/ml) was inactivated in a water bath at 65°C for 60 min, as well as the following: 50 μl of inactive IAP and 50 μl of LPS (2.0 ng/ml); 50 μl of IAP (40 U/ml) + 50 μl of ATP/UTP/GTP/CTP/TTP (4.0 mmol/l); 50 μl of IAP (2/10/20/40 U/ml), and 50 μl of ATP (4.0 mmol/L); 100 μl of ATP, ADP, AMP, and adenosine (YuanYe Biotechnology Co., Ltd., China) at final concentrations of 0.10, 0.25, 0.50, and 1.00 mmol/l; 50 μl of IAP (40 U/ml) and 50 μl of IAP (40 U/ml) + 50 μl of CCPA, DPCPX, CGS21680, DMPX, NECA, MRS1754, Cl-IB-MECA, and MRS1220 (4–40,000 nmol/l, Sigma, USA); and 100 μl of uridine, guanosine, cytidine, thymidine (YuanYe Biotechnology Co., Ltd., China). Additionally, 100 μl of adenosine (2.0 mmol/L) and 50 μl of adenosine (2.0 mmol/l) + 50 μl of uridine, guanosine, cytidine, and thymidine (2.0 mmol/l) were added at final concentrations of 0.10, 0.25, 0.50, and 1.00 mmol/l. All groups were added to the same cell line and cultured at 37°C for 24 h.

### Determination of pro-inflammatory cytokines

Supernatants were collected, and TNF-α and IL-6 levels were determined using ELISA (R&D Systems, UK).

The supernatant was collected after LPS administration, with or without IAP, adenosine, adenosine + uridine, guanosine, cytidine, and thymidine pretreatment. Then, 50 μl of the supernatant in each well was absorbed into blank plate wells. Afterwards, 50 μl of Griess A (5% H_3_PO_4_ solution containing 1% sulfa) and Griess B (1% *N*-1-naphthyl-ethylenediamine-hydrochloric acid solution) were added, and the OD value of each well was measured at 570 nm. PGE_2_ was determined using a prostaglandin E_2_ assay kit (H099-1, Nanjing Jiancheng Biotechnology Co., Ltd., China) (25).

### Cell viability

The control group was treated with 100 μl of RPMI-1640 complete medium, and the experimental group was treated with 100 μl of LPS (1.0 ng/ml), 100 μl of IAP (40 U/ml), and 50 μl IAP (4/20/40/80 U/ml) + 50 μl LPS (2.0 ng/ml). Additionally, 100 μl of adenosine (2.0 mmol/l) and 50 μl adenosine (2.0 mmol/l) + uridine, guanosine, cytidine, and thymidine (2.0 mmol/l) were added to the cell lines and cultured at 37°C for 24 h. The supernatant was discarded, and adherent cells were cleaned with PBS. Each well was added with 180 μl of serum-free medium and 20 μl of CCK8 (Beyotime, China) and cultured for 4 h. The OD of each well was measured at 450 nm. Survival rate (%) was calculated as follows: survival rate = (OD test group/OD blank group) × 100% (26).

### Cell morphology

Control, LPS, IAP, IAP + LPS, adenosine, and adenosine + uridine, guanosine, cytidine, and thymidine were added in a 24-well plate in triplicates. The morphological characteristics of the cells were observed under an inverted phase-contrast microscope (OLYMPUS, Japan) at 0, 24, and 36 h.

### Wound healing assay

Control, LPS, IAP, IAP+LPS, adenosine, and adenosine + uridine, guanosine, cytidine, and thymidine were seeded in a six-well plate. Scratches were generated using a 1-ml micropipette tip when the cells reached 100% confluence. The cells were then washed twice with PBS and incubated in a complete medium at 37°C. Images were observed under an inverted phase-contrast microscope (OLYMPUS, Japan) after 0, 48, and 72 h. Cell areas were quantified using ImageJ, and cell migration was defined as [Cell area (48/72 h)–Cell area (0 h)/Cell area (0 h)] (27).

### ATP, ADP, AMP, and adenosine release

The supernatant was collected 20 min after LPS administration, with or without IAP pretreatment. ATP, ADP, AMP, and adenosine production were directly measured using the Bioluminescence Assay Kit CLS II (Mlbio, China).

### RNA sequencing and data analysis

HT-29 cells were inoculated on six-well plates (4.0 × 10^4^ cells/well) in the presence of 5% CO_2_ at 37°C for 24 h. The supernatant was removed, and 1 ml of freshly extracted leucocytes (2 × 10^5^ cells/well) was added. The control group was supplemented with 1 ml of R1640 medium, and the LPS group was added with 1 ml of LPS (1.0 ng/ml). The IAP + LPS group was added with 500 μl of IAP (40 U/ml) and 500 μl of LPS (0.5 ng/ml). The adenosine group was added with 1 ml of adenosine (2 mmol/l). Plates were then centrifuged, and supernatants were discarded. Total RNA was extracted from the mycelia by using TRIzol reagent (Sigma, USA). The quantity and purity of the RNA were assessed *via* 1% agarose gel electrophoresis and NanoDrop 2000 (Thermo Fisher Scientific, MA, USA). RNA was quantified using a Qubit3.0, Agilent 2100, StepOnePlus™ Real-Time PCR System (Thermo Fisher Scientific, MA, USA). RNA integrity was examined with an RNA Nano 6000 assay kit of the Bioanalyser 2100 system (Agilent Technologies, CA, USA).

### Quantitative real-time PCR

Total RNA was extracted from the mouse jejunum tissue and cell lines by using the TRIzol reagent. cDNA was diluted to 2 ng/µl for quantitative PCR. The primers were designed using Primer3.0. Then, 2× SYBR^®^ Green premix was used in the reaction system. **Table S2** shows the real-time fluorescence quantitative PCR system, and **Table S3** presents the real-time fluorescence quantitative PCR. The program was set as follows: 95°C, 5 min → 95°C, 15 s → 60°C × 30 s; the whole process was 40 cycles. The relative expression levels of differential genes were analysed with 2^-ΔΔCt^.

### Statistical analysis

Data were statistically analysed using SPSS, expressed as mean ± SD, and examined using two-tailed unpaired Student’s t test. Statistically significant differences between more than two test groups were evaluated *via* one-way analysis of variance with Tukey’s multiple-comparison posttests. Differences were considered significant when P < 0.05.

## Results

### Dephosphorylation properties of IAP

**Figure 1:**
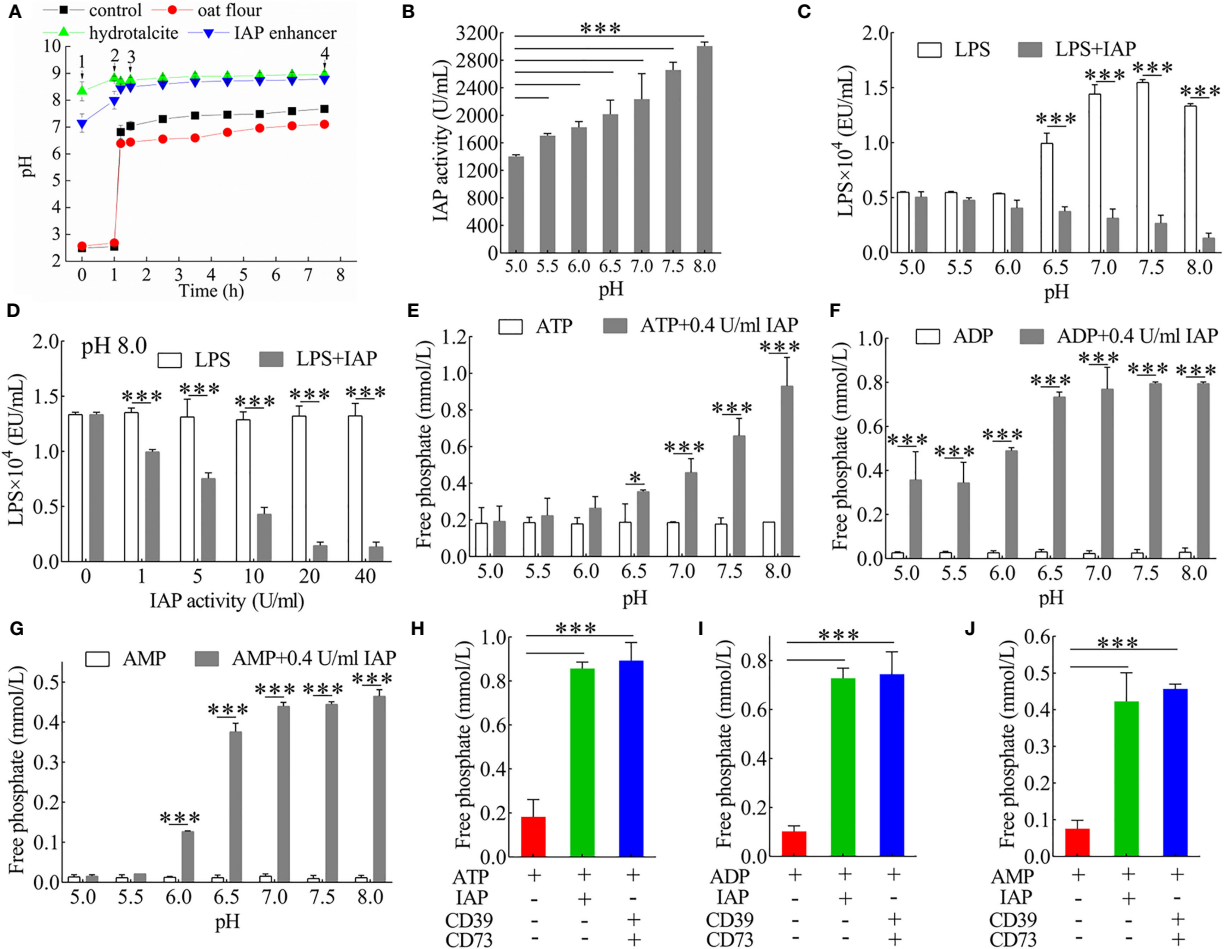
Analysis of the dephosphorylation properties of IAP. **(A)** Buffering capacity of IAP enhancer, hydrotalcite, and oat flour during gastrointestinal stimulation *in vitro*. 1) Into the stomach; 2) into the duodenum; 3) into the small intestine; 4) into the large intestine. **(B)** The pH-dependent activity of IAP. The inactivation effect of IAP on LPS at **(C)** different pH levels and **(D)** different IAP activities. The dephosphorisation of **(E)** ATP, **(F)** ADP, and **(G)** AMP by 0.4 U/ml IAP at different pH levels. Dephosphorylation of **(H)** ATP, **(I)** ADP, and **(J)** AMP by IAP or CD39 + CD73. Values are presented as means ± SD (n = 3/group). Two-tailed unpaired Student’s t test. For multiple comparisons, analysis of variance with Tukey was used. *P < 0.05; ***P < 0.001.

We first examined the dephosphorylation of IAP on LPS or nucleoside triphosphate *in vitro* to reduce the intestinal inflammatory response and prevent IR caused by intestinal inflammation. We simulated the slow-release effects of the IAP enhancer in a simulated gastrointestinal environment. When the IAP enhancer and hydrotalcite mixture interacted with the artificial stomach acid, both exhibited a good slow-release ability and could not be resolved by the acid. Consequently, it could reach the duodenum and small intestine and exhibit a good slow-release ability to increase the pH of the small intestine *in vitro* (**Figure 1A**). Thus, the IAP activity improved. We further demonstrated that increasing pH was an effective way to enhance IAP activity *in vitro* (**Figure 1B**).

LPS induces inflammatory factors that interfere with insulin signalling and contribute to IR (28). It releases inorganic phosphate through the IAP dephosphorylation (12). LPS was dephosphorylated by IAP in a pH- (**Figure 1C**) and dose-dependent manner (**Figure 1D**) *in vitro*. The results were further investigated to determine whether IAP could dephosphorylate nucleoside triphosphate *in vitro*. IAP could further dephosphorylate ATP (**Figure 1E**) and its products ADP (**Figure 1F**) and AMP (**Figure 1G**) in a pH-dependent manner; however, when the activity was high enough, pH slightly affected dephosphorylation (**Figure S1**). IAP dephosphorylation was compared with the hydrolase CD39 and CD73 of ATP, ADP, and AMP. As expected, the effect of IAP was similar to that of CD39 and CD73, which dephosphorylated ATP (**Figure 1H**), ADP (**Figure 1I**), and AMP (**Figure 1J**) to adenosine. Furthermore, IAP dephosphorylation was dependent on pH for other nucleoside triphosphates such as UTP, UDP, and UMP (**Figure S2**); GTP, GDP, and GMP (**Figure S3**); CTP, CDP, and CMP (**Figure S4**); and TTP, TDP, and TMP (**Figure S5**). Therefore, pH had an undecisive role in these ligands during a high IAP activity (**Figures S6–S9**).

### Intervention effect of IAP and IAP enhancer in mouse models

**Figure 2:**
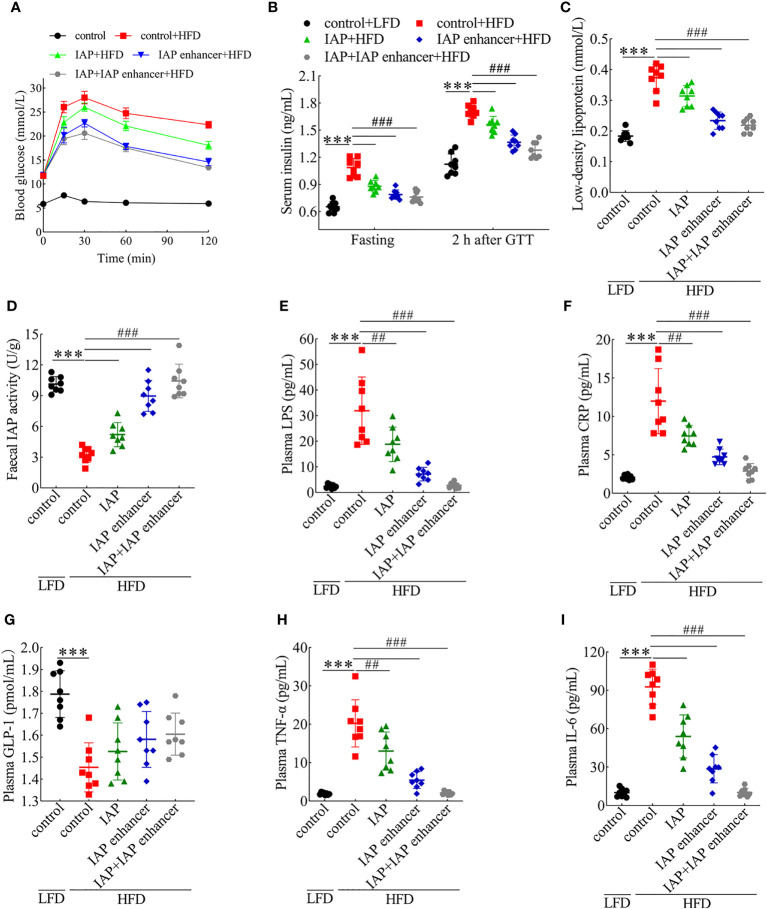
Preventive effects of IAP + IAP enhancer on HFD-induced glucose intolerance and IR. **(A)** Oral glucose tolerance. **(B)** Fasting and postprandial insulin. **(C)** Low-density lipoprotein. **(D)** Faecal IAP. **(E)** Plasma LPS. **(F)** Plasma CRP. **(G)** Plasma GLP-1. **(H)** Plasma TNF-α. **(I)** Plasma IL-6. Values are presented as means ± SD (n = 8/group). Two-tailed unpaired Student’s t test. HF control groups compared with the LF control groups, ***P < 0.001; HF treatment groups compared with the HF control groups, ^##^P < 0.01, ^###^P < 0.001.

We next investigated whether IAP and IAP enhancer could reverse any features of IR. We observed a higher body weight in the HFD group than in the other groups; however, IAP and IAP enhancer prevented the HFD-induced increase in body weight especially in the IAP + IAP enhancer group (**Figure S10**). Compared with the vehicle, the oral glucose tolerance of the mice treated with IAP + IAP enhancer was better (**Figure 2A** and **Figure S11**) than that of the mice administered with either IAP or IAP enhancer. The lowest fasting and postprandial insulin was observed in the IAP + IAP enhancer group (**Figure 2B**). Cholesterol, triglyceride, and high-density lipoprotein did not significantly vary among the three groups (**Figures S12A-C**). Low-density lipoprotein levels were the most significant in the IAP + IAP enhancer group (**Figure 2C**). Based on the IAP activity influenced by pH, the IAP activity was increased by increasing the intestinal pH *in vivo*. Urine pH was improved in the IAP enhancer and IAP + IAP enhancer groups, reaching the urine pH of the LFD control group (**Figure S12D**). This result was consistent with our hypothesis that urine pH was positively correlated with intestinal pH. As such, we measured the IAP activity in the faecal sample. The HFD-fed mice had less faecal IAP activity than the LFD control group, but the faecal IAP activity of the HFD-fed mice increased significantly after the treatment, especially in the IAP + IAP enhancer group (**Figure 2D**). Plasma LPS levels were much higher in the HFD-fed mice than in the LFD control group, but these levels were reduced after the treatment (**Figure 2E**). These results supported the concept that IAP prevented LPS activity by detoxifying LPS within the intestinal lumen (12). The plasma CRP level significantly decreased in the three groups; among them, the IAP + IAP enhancer group had the best effect on reducing the inflammation level. However, none of the three groups could reach the level of the LFD control group after intervention (**Figure 2F**). IAP, IAP enhancer, and IAP + IAP enhancer significantly increased the plasma GLP-1 levels, and their effects exhibited the following trend: IAP < IAP enhancer < IAP + IAP enhancer (**Figure 2G**). The three intervention groups could promote GLP-1 secretion by intestinal mucosa L cells and reduce blood glucose, which affects IR (29). To further assess the local inflammatory status within the intestine, we measured plasma TNF-α and IL-6 levels and found that the levels of HFD mice were significantly higher than those of the LFD control group (**Figures 2H, I**); those treated with IAP + IAP enhancer showed the lowest levels.

**Figure 3:**
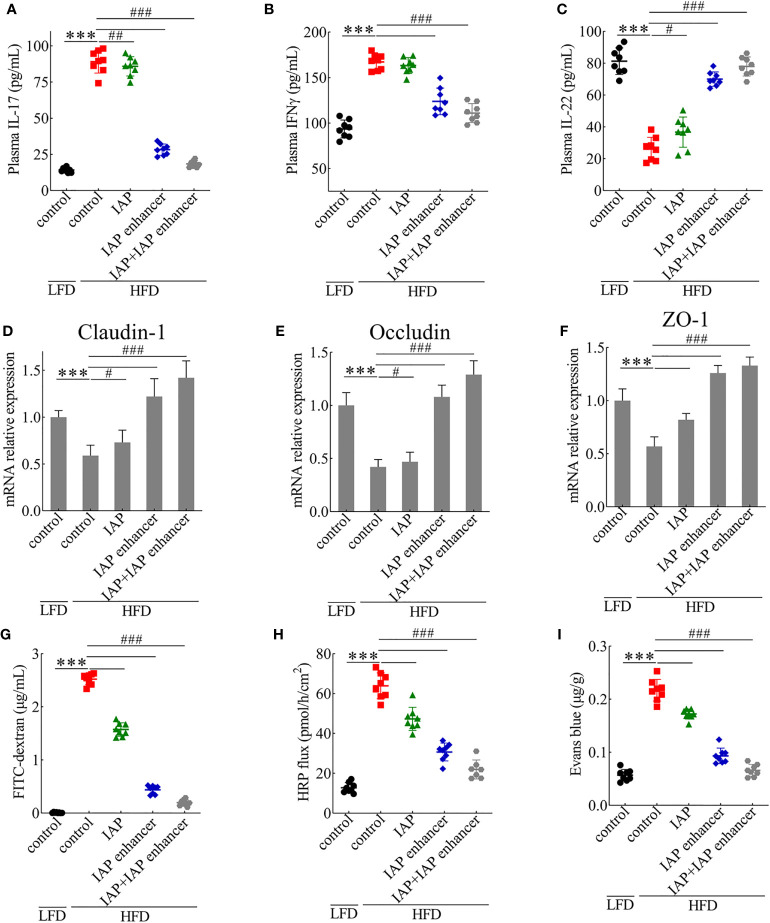
IAP + IAP enhancer prevents HFD-induced intestinal permeability injury. **(A)** IL-17. **(B)** IFNγ. **(C)** IL-22. The relative mRNA expression levels **(D)** claudin-1, **(E)** occludin, and **(F)** ZO-1. Intestinal permeability as determined by **(G)** FITC-dextran (70 kDa), **(H)** HRP flux ex vivo, and **(I)** Evans blue *in vivo* after oral gavage. Values are presented as means ± SD (n = 8/group). Two-tailed unpaired Student’s t test. HF control groups compared with the LF control groups, ***P < 0.001; HF treatment groups compared with the HF control groups, ^#^P < 0.05, ^##^P < 0.01, ^###^P < 0.001.

IL-17, IFNγ, and IL-22 alter tight junction activity (30). As depicted, serum IL-17 and IFNγ concentrations significantly increased, whereas IL-22 concentrations decreased in the HFD mice. Compared with those in the IAP and IAP enhancer, IAP + IAP enhancer treatment significantly reduced the concentrations of IL-17 and IFNγ (**Figures 3A, B**) but increased the concentration of IL-22 (**Figure 3C**). These results indicated that IAP + IAP enhancer could restore intestinal barrier function by controlling relevant cytokines *in vivo*.

Intrigued by these results, we examined the effect of IAP + IAP enhancer on the jejunum epithelium of HFD-fed mice with disrupted intestinal barrier functions. HFD mice orally administered with IAP + IAP enhancer had normal expression patterns and mRNA levels of claudin-1 (**Figure 3D**), occludin (**Figure 3E**), and ZO-1 (**Figure 3F**), which are tight junction-associated proteins that play pivotal roles in gut homeostasis (31). Furthermore, in comparison with other treatments, IAP + IAP enhancer remarkably prevented the systemic exposure of FITC-dextran after oral administration in the HFD model group (**Figure 3G**), demonstrating the restoration of intestinal barrier functions. The HRP flux more significantly decreased in the IAP + IAP enhancer group than in the other groups (**Figure 3H**). When the intestinal contents were tested with Evans blue, the colour was lighter in the IAP + IAP enhancer than in the other treatments (**Figure 3I**).

**Figure 4:**
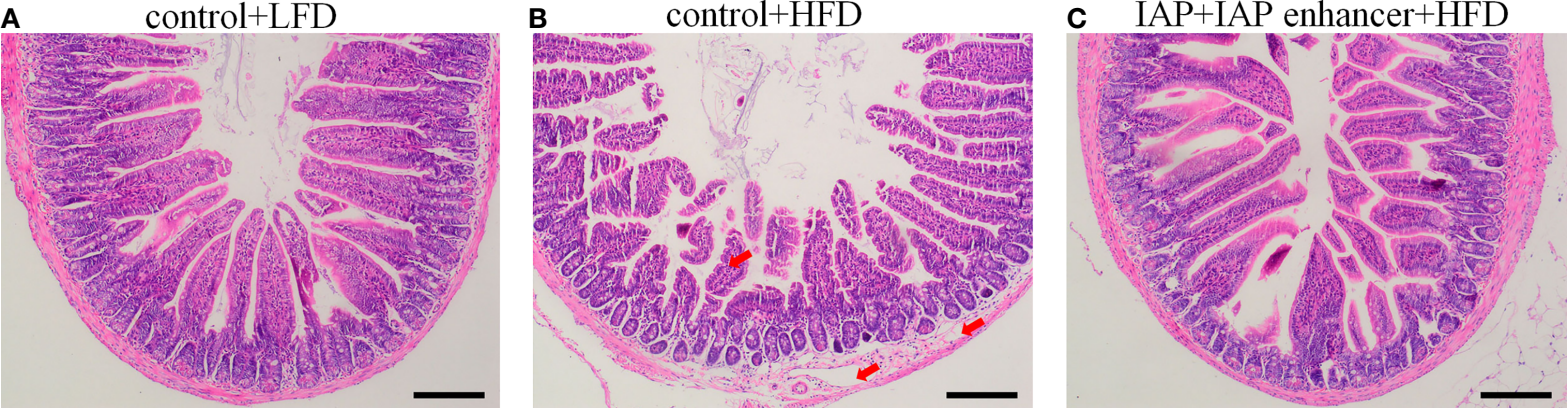
Effect of IAP + IAP enhancer treatment on jejunum histopathological alterations (H&E staining, magnification ×100). **(A)** control + LF group; **(B)** control + HF group; **(C)** IAP + IAP enhancer group. The arrows point to the HFD group, and the villi were shortened; the lamina propria was oedematous.

Histological observation showed no damage on the jejunum of the control + LFD group (**Figure 4A**), but the jejunum structure of the control + HFD group was damaged, and the villi were broken and scattered (**Figure 4B**). However, the jejunum structure of the IAP + IAP enhancer group was significantly improved compared with that of the control + HFD group, and the villi were arranged neatly (**Figure 4C**).

### Anti-inflammatory mechanism of IAP *in vitro*

**Figure 5:**
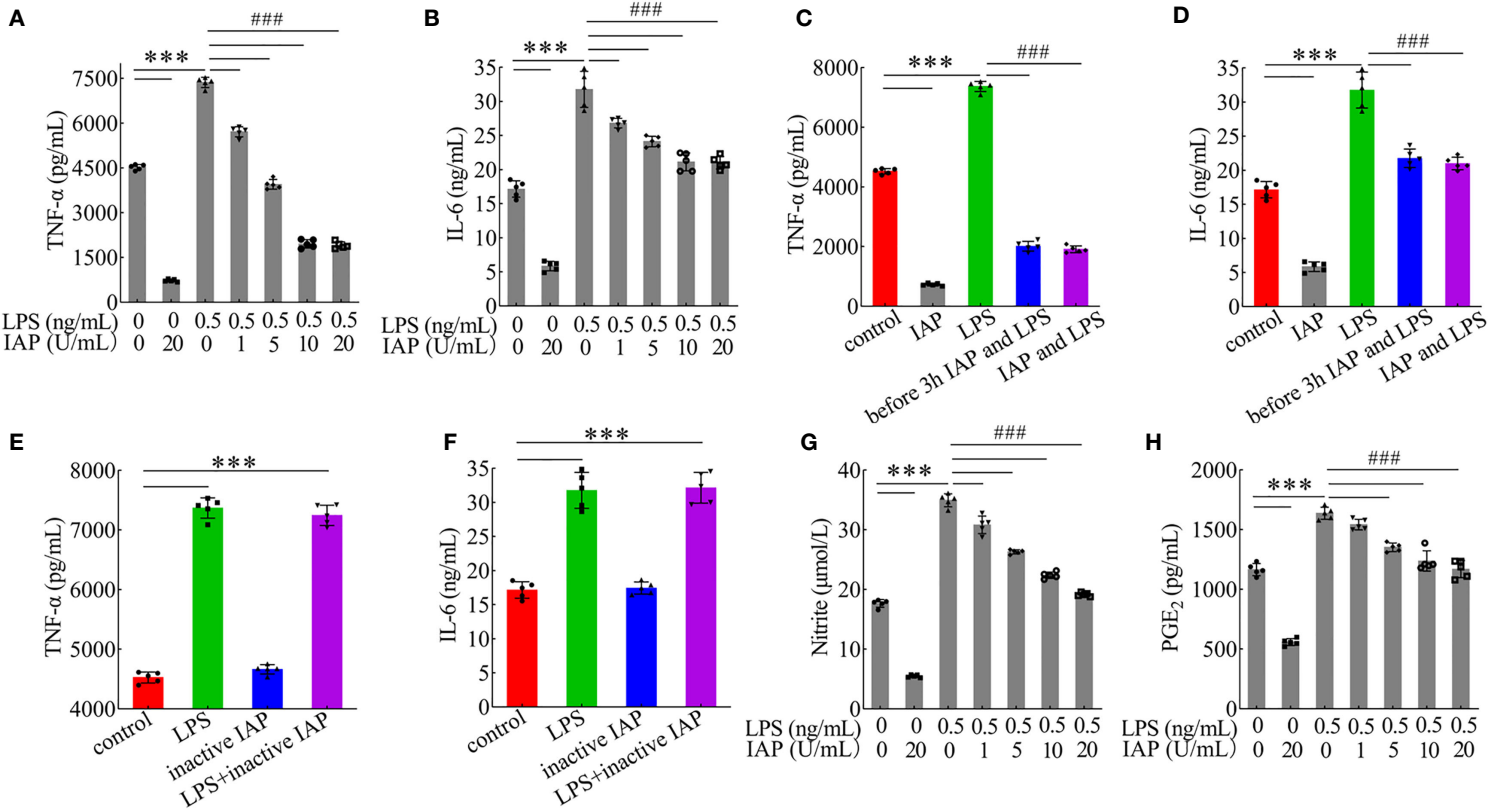
Effect of IAP in the inflammatory response induced by LPS. The effect of different IAP activity on LPS-induced **(A)** TNF-α and **(B)** IL-6 in cocultured HT-29 cells + freshly extracted human leucocytes. Different IAP and LPS incubation methods (IAP and LPS were added after incubation for 3 h, or IAP and LPS were added simultaneously) on the secretion of **(C)** TNF-α and **(D)** IL-6 in cocultured HT-29 cells + freshly extracted human leucocytes. Inactivation of IAP on LPS induced **(E)** TNF-α and **(F)** IL-6 in cocultured HT-29 cells + freshly extracted human leucocytes. The effect of different IAP activity on LPS-induced **(G)** NO and **(H)** PGE_2_ in cocultured HT-29 cells + freshly extracted human leucocytes. Values are presented as means ± SD (n = 5 wells/group). Analysis of variance with Tukey. Compared with the control, ***P < 0.001, compared with the LPS, ^###^P < 0.001.

HT-29 cells + freshly extracted human leucocytes were used to establish a model of TNF-α and IL-6 produced by LPS stimulation. The LPS concentration was 0.5 ng/ml, and the TNF-α (**Figure S13A**) and IL-6 (**Figure S13B**) contents continuously increased until the maximum values were reached. Moreover, with the participation of leucocytes, more TNF-α/IL-6 could be produced in the coculture system even without treatment. IAP could inhibit TNF-α and IL-6 in the model with or without LPS. The results suggested that IAP could reduce inflammation *via* another anti-inflammatory pathway other than LPS inactivation *in vitro*. When the LPS concentration was 0.5 ng/ml, the reduction of TNF-α (**Figure 5A**) and IL-6 (**Figure 5B**) showed a dose-dependent relationship with IAP activity. A similar protective result was obtained when the IAP was incubated with LPS for 3 h in advance or simultaneously (**Figures 5C, D**). The effect of inactive IAP lacking its hydrolysing properties was investigated to confirm that the IAP-induced reduction in cytokine production was due to the dephosphorylating nature of the enzyme. Inactive IAP did not attenuate the LPS-induced inflammatory response in the model (**Figures 5E, F**). Additionally, IAP inhibited the inflammatory markers NO (**Figure 5G**) and PGE_2_ (**Figure 5H**) in a dose-dependent manner to further assess the inflammatory status within the intestine.

The proliferation, differentiation, and survival of intestinal epithelial cells are regulated by cytokines, such as TNF-α and IL-6 (32). Therefore, the inflammation degree was affected by HT-29 cell proliferation. IAP could significantly inhibit cell proliferation, while LPS could significantly promote cell proliferation. When IAP was co-incubated with LPS, inhibition was positively correlated with IAP dose (**Figure S14**).

**Figure 6:**
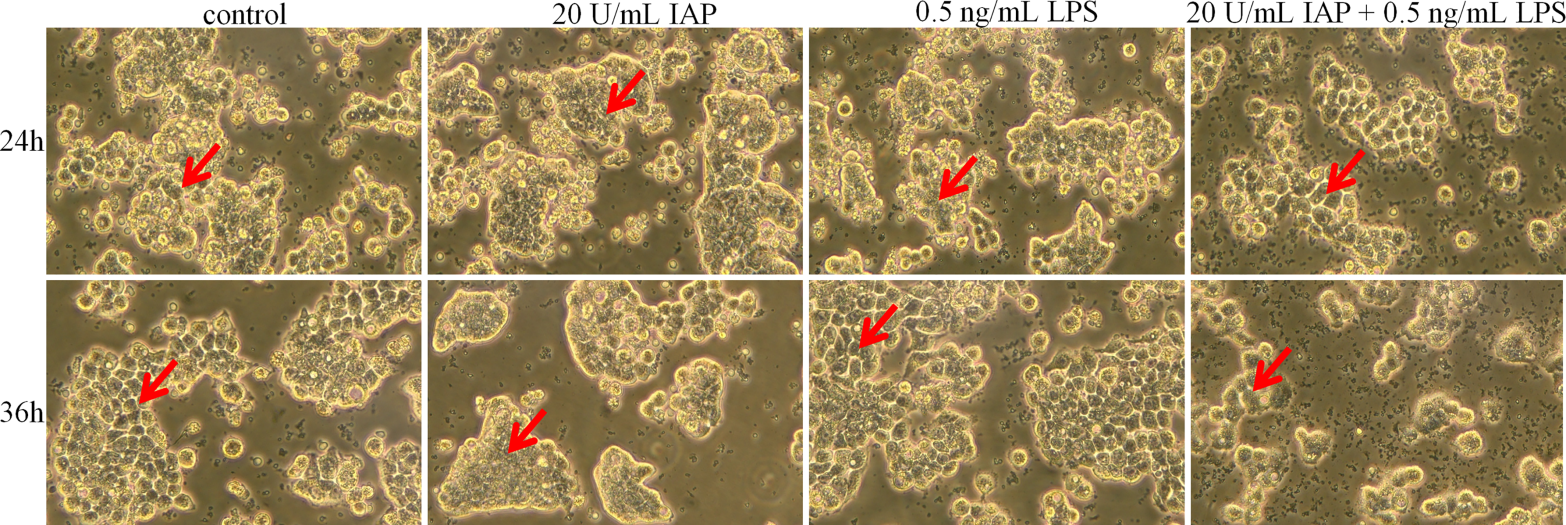
Effect of IAP on LPS-induced cell morphology in co-cultured HT-29 cells + freshly extracted human leucocytes. Cell morphology was obtained by taking bright field images with an OLYMPUS inverted microscope (×100). All experiments were repeated at least thrice, and a representative figure is displayed. The arrows point to the observation areas of focus.

After 24 h, the control group and the experimental group did not have noticeable morphological changes. After 36 h, the control group cells showed an irregular shape, large volume, and small intercellular space. The cell volume gradually decreased, and the cells became round; the cell wall was not firm. After LPS treatment, cell proliferation was induced, resulting in tighter intercellular connections. When treated with IAP + LPS, the number of cells decreased, and the shape of cells gradually enlarged. The dead cells and debris were suspended (**Figure 6**).

**Figure 7:**
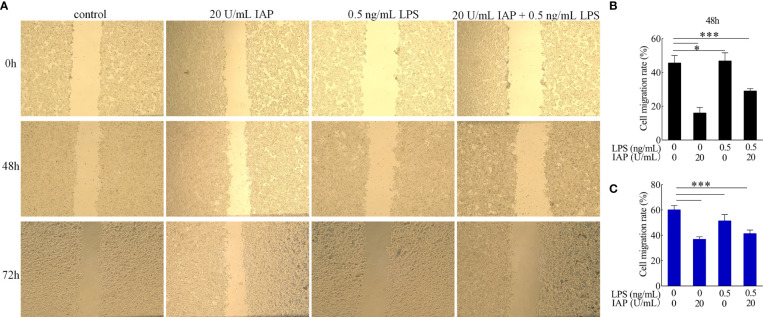
Effect of IAP on LPS-induced **(A)** cell migration and cell migration rate of **(B)** 48 and **(C)** 72 h in cocultured HT-29 cells + freshly extracted human leucocytes. Images was obtained by an OLYMPUS inverted microscope (×20). Values are presented as means ± SD (n = 3/group). Analysis of variance with Tukey. *P < 0.05; ***P < 0.001.

Cytomorphogenesis and inflammation were evaluated in terms of cell migration (33). IAP significantly inhibited the migration of HT-29 cells at 48 and 72 h compared with that of the control group. After LPS treatment, HT-29 cell migration was significantly stimulated. Cell migration was significantly reduced after co-incubation with LPS and IAP at 48 and 72 h (**Figure 7**).

**Figure 8:**
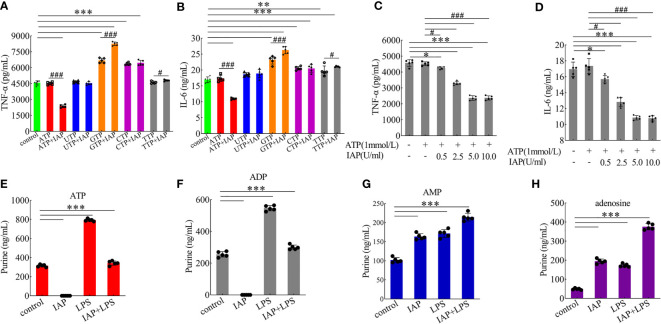
Anti-inflammatory effect of IAP on dephosphorylation of nucleoside triphosphate. The effect of IAP on the secretion of **(A)** TNF-α and **(B)** IL-6 by dephosphorylation of nucleoside triphosphate. The effect of different IAP activity on ATP dephosphorylation on the secretion of **(C)** TNF-α and **(D)** IL-6. The effect of IAP and LPS on **(E)** ATP, **(F)** ADP, **(G)** AMP, and **(H)** adenosine release in cocultured HT-29 cells + freshly extracted human leucocytes. Values are presented as means ± SD (n = 5 wells/group). Two-tailed unpaired Student’s t test. For multiple comparisons, analysis of variance with Tukey was used. **(A, B)** compared with control, *P < 0.05, **P < 0.01,***P < 0.001; compared with the nucleoside triphosphate group, ^#^P < 0.05, ^###^P < 0.001. **(E–H)** ***P < 0.001.

Based on the known IAP activity and our data on luminal LPS, our results suggested that IAP may prevent IR through another mechanism that involves the luminal dephosphorylation of ATP and other substances associated with inflammation except LPS dephosphorylation (34). We tested five IAP targets, namely, ATP, UTP, GTP, CTP, and TTP, and discovered that UTP, GTP, CTP, and TTP had no significant tendency on cytokines after dephosphorylation by IAP. However, ATP significantly changed the TNF-α (**Figure 8A**) and IL-6 (**Figure 8B**) levels. Furthermore, the TNF-α and IL-6 levels increased significantly after GTP and TTP dephosphorylation by IAP (**Figures 8A, B**).

The results showed the anti-inflammatory effect of the different activities of IAP on ATP dephosphorylation and found that TNF-α (**Figure 8C**) and IL-6 (**Figure 8D**) were inhibited by ATP dephosphorylation in a dose-dependent manner *in vitro*. On this basis, we examined the anti-inflammatory effects of ATP and its dephosphorylated products ADP, AMP, and adenosine. ATP (**Figures S15A, B**) has no anti-inflammatory function. However, ADP (**Figure S15C, D**) and AMP (**Figures S15E, F**) had a significant anti-inflammatory effect; the inhibitory effect of adenosine (**Figures S15G, H**) was the most significant.

**Figure 9:**
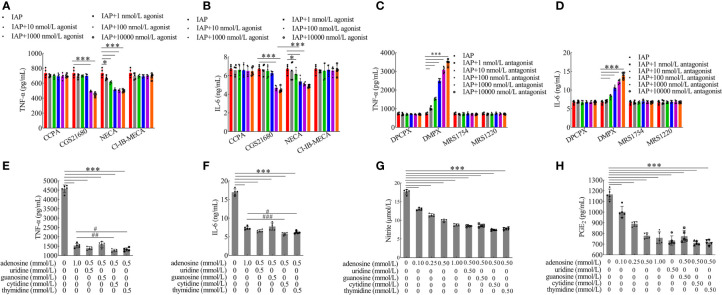
Anti-inflammatory effect of adenosine receptors and nucleoside synergies. The effect of adenosine receptor **(A, B)** agonist and **(C, D)** antagonist on the secretion of TNF-α and IL-6 produced in co-cultured HT-29 cells + freshly extracted human leucocytes. The effect of adenosine and other nucleosides on **(E)** TNF-α, **(F)** IL-6, **(G)** NO, and **(H)** PGE_2_ in cocultured HT-29 cells + freshly extracted human leucocytes. Values are presented as means ± SD (n = 5 wells/group). Analysis of variance with Tukey. **(A–D)** *P < 0.05; ***P < 0.001. **(E–H)** compared with the control, ***P < 0.001; compared with the adenosine group (1 mmol/l), ^#^P < 0.05, ^##^P < 0.01, ^###^P < 0.001. CCPA: A_1_ receptor agonist, CGS21680: A_2A_ receptor agonist, NECA: A_2A_ and A_2B_ receptor agonist, CI-IB-MECA: A_3_ receptor agonist, DPCPX: A_1_ receptor antagonist, DMPX: A_2A_ receptor antagonist, MRS1754: A_2B_ receptor antagonist, MRS1220: A_3_ receptor antagonist.

Extracellular ATP can be converted by ectonucleotidases into AMP and eventually into adenosine. Interestingly, this study showed that extracellular ATP and ADP concentrations increased after LPS incubation, but this increase was reversed by IAP pre-incubation. These concentrations respectively increased by 251.85% and 117.28% relative to the control after LPS stimulation; after the co-administration of IAP, these concentrations increased by 108.94% and 117.28%, respectively. Subsequently, IAP directly converted ATP and ADP into AMP and adenosine (**Figures 8E–H**). Therefore, adenosine was produced through the IAP dephosphorylation of ATP, ADP, and AMP. A_2A_ and A_2B_ could be the receptors of action (**Figures 9A, B**). As shown in **Figures 9C, D**, adenosine inhibited TNF-α and IL-6 through the A_2A_ receptor in this model.

Although uridine and cytidine had anti-inflammatory effects, they were unstable and mainly restricted by dose condition; guanosine and thymidine had no anti-inflammatory function (**Figure S16**). However, adenosine combined with cytidine and thymidine had significant synergistic effects (**Figures 9E, F**). Compared with the control group, adenosine combined with other nucleosides had extremely significant inhibitory effects on TNF-α (**Figures 9E**) and IL-6 (**Figure 9F**). Moreover, adenosine inhibited the concentrations of NO (**Figure 9G**), PGE_2_ (**Figure 9H**), and proliferating cells (**Figure S17**) in a dose-dependent manner.

**Figure 10:**
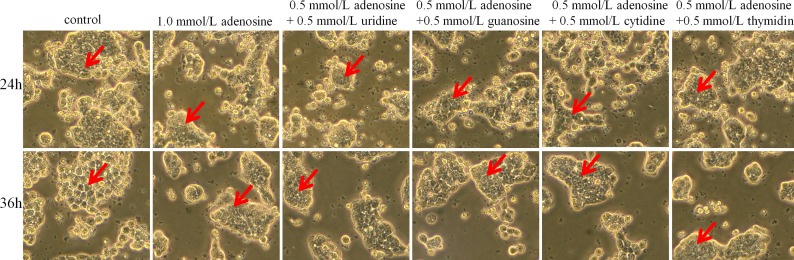
Effect of adenosine and other nucleosides on cell morphology in cocultured HT-29 cells + freshly extracted human leucocytes. Cell morphology was obtained by taking bright field images with OLYMPUS inverted microscope (×100). All experiments were repeated at least three times, and a representative figure is displayed. The arrows point to observation areas of focus.

As shown in **Figure 10**, cell morphology did not significantly differ after 24 h. However, after 36 h, the cells in the adenosine and adenosine combined with other nucleoside groups showed increased roundness, significantly decreased adhesion ability, and had a better dispersion state compared with those in the control group. Conversely, the control group showed giant tumour cells and more fibroblast-like cells, scattered or connected by the cytoplasm. The cells adhered firmly to the wall and grew well.

**Figure 11:**
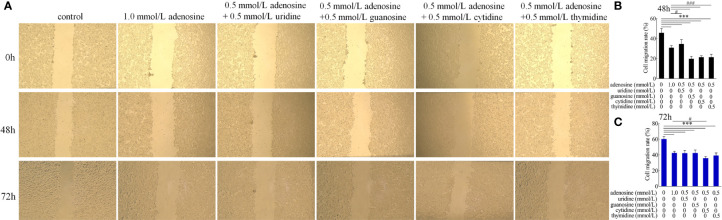
Effect of adenosine and other nucleosides on **(A)** cell migration and cell migration rate of **(B)** 48 and **(C)** 72 h in cocultured HT-29 cells + freshly extracted human leucocytes. Images were obtained by an OLYMPUS inverted microscope (×20). Values are presented as means ± SD (n = 3/group). Analysis of variance with Tukey. compared with the control, ***P < 0.001; compared with to the adenosine group, ^#^P < 0.05; ^###^P < 0.001.

For cell migration, adenosine combined with guanosine, cytidine, and thymine had a synergistic inhibitory effect compared with that of the adenosine group at 48 h. At 72 h, adenosine combined with cytidine only elicited a synergistic inhibitory effect (**Figure 11**).

### Transcriptome analysis of the anti-inflammatory effect of IAP *in vitro*

To better understand the anti-inflammatory mechanisms of IAP to prevent IR by suppressing inflammation *in vitro*, we conducted a comparative transcriptomic analysis of the control, LPS, IAP + LPS, and adenosine groups. We found that 77 DEGs were upregulated and 193 DEGs were downregulated among the 270 DEGs in IAP + LPS vs. LPS (**Figure S18A**). Furthermore, 93 DEGs were upregulated and 244 DEGs were downregulated among the 337 DEGs in IAP + LPS vs. control (**Figure S18B**). In addition, 489 and 743 DEGs were upregulated or downregulated among the 1,232 DEGs in adenosine vs. control (**Figure S18C**). The DEGs between the sample groups were then visualised using hierarchical clustering diagrams (**Figure S19**).

GO enrichment analysis indicated the biological process significantly associated with DEGs among IAP + LPS vs. LPS (**Figure S20A**), IAP + LPS vs. control (**Figure S20B**), and adenosine vs. control (**Figure S20C**).

**Figure 12:**
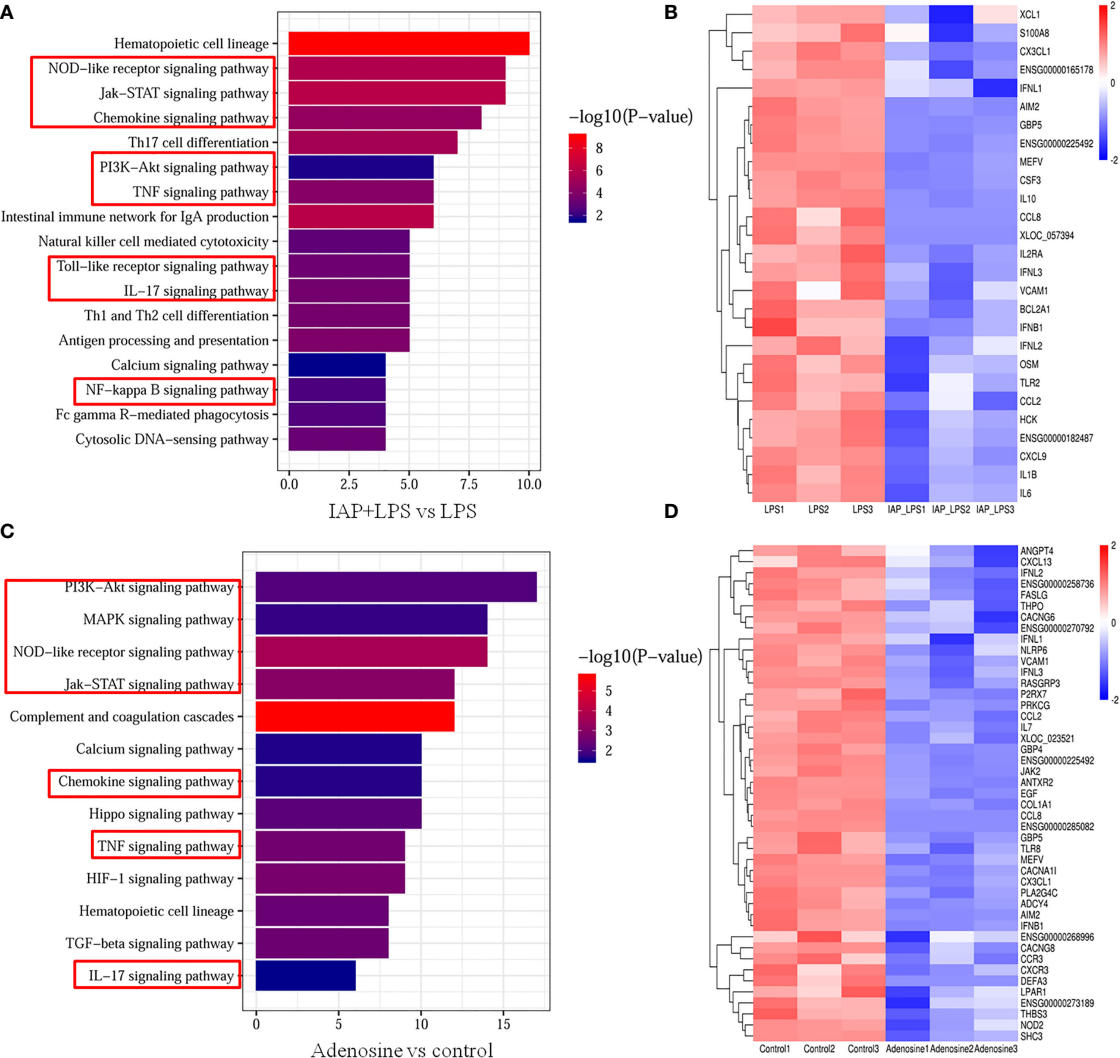
Transcriptome analysis of the anti-inflammatory effect of IAP *in vitro*. Signalling pathways related to the immune system and signal transduction KEGG enrichment of **(A)** IAP+LPS vs. LPS and **(C)** adenosine vs. control. Expression heatmap of signature genes in intestinal inflammation of **(B)** IAP+LPS vs. LPS and **(D)** adenosine vs. control.

KEGG database analysis revealed differences in 81 pathways (P < 0.05) in the IAP + LPS vs. LPS. Of the total differential KEGG pathways between IAP + LPS and LPS groups, 17 signalling pathways that were closely related to the immune system and signal transduction were selected (P < 0.05), and eight of these pathways were associated with intestinal inflammation, TNF, chemokine, Toll-like receptor, PI3K-Akt, JAK-STAT, IL-17, NOD-like receptor, and NF-κB signalling pathways (**Figure 12A**). The corresponding genes are shown in **Figure 12B**. CCL2 (35) and CX3CL1 (36) are essential cytokines in the TNF signalling pathway. CXCL9 (37) and CX3CL1 (38) can inhibit the chemokine signalling pathway. The expression levels of IL1B (39), IL-6 (40), and IFNB1 (41) significantly increased in IAP + LPS vs. LPS, which has a vital role in the Toll-like receptor signalling pathway. In the PI3K-Akt signalling pathway, the expression levels of the critical regulatory factor CXCL9 (42) were significantly lower in the IAP + LPS group than in the LPS group. The expression levels of the Jak-STAT signalling pathway (IFNL1, IFNL2, and IFNL3) (43) were significantly lower in the IAP + LPS group than in the LPS group. The IL-17 signalling pathway involves some important regulatory molecules, such as CSF3 (44) and CCL2 (45), which were significantly different between the IAP + LPS and LPS groups. In the NOD-like receptor signalling pathway, the expression levels of HCK (46) were significantly lower in the IAP + LPS group than in the LPS group. The NF-κB signalling pathway showed a downward trend in the IAP + LPS group, and the expression levels of MEFV (47) and S100A8 (48) significantly decreased. The associated pathways in IAP + LPS vs. control were similar to those in IAP + LPS vs. LPS (**Figure S21**).

In comparison with the control group, 72 signalling pathways were identified after adenosine treatment (P < 0.05). Furthermore, 13 immune systems were screened in signal transduction pathways (P < 0.05) of which seven were closely related to intestinal inflammation: TNF, chemokine, NOD-like receptor, PI3K-Akt, Jak-STAT, IL-17, and MAPK signalling pathways (**Figure 12C**). As shown in **Figure 12D**, the same gene of the IAP + LPS group relevant for the TNF signalling pathway was downregulated in the adenosine group, indicating that the TNF signalling pathway is a potential inflammatory pathway in the adenosine group compared with that in the control group. CXCL13 (37), CXCR3 (37), CX3CL1 (49), and CCR3 (50), which participate in the chemokine signalling pathway were significantly downregulated after adenosine treatment. In the PI3K-Akt signalling pathway, LPAR1 (51), SHC3 (52), and EGF (53) were downregulated in the adenosine group. IFNL1, IFNL 2, IFNL 3 (43), and THPO (54) were downregulated, suggesting that the Jak-STAT signalling pathway was affected by adenosine. CSF3 (44), CCL2 (45), and TRAF6 (55) were also significantly inhibited, implying that the IL-17 signalling pathway was also involved in the suppression of intestinal inflammation by adenosine. Meanwhile, the HCK gene expression of the NOD-like receptor signalling pathway decreased in the adenosine group (46). Several genes, including THPO (54), PLA2G4C (56), and FASLG (57), relevant to the MAPK signalling pathway were significantly downregulated. Therefore, understanding the role of IAP and adenosine in inhibiting intestinal inflammation pathways may help inhibit intestinal inflammation by controlling the targeted genes, thereby preventing IR. The comparison of the transcriptome data with the qRT-PCR results showed that qRT-PCR and RNA-Seq revealed similar up- or downregulation patterns for these genes, thereby validating the RNA-Seq data (**Figure S22**).

## Discussion

In this study, we developed an IAP + IAP enhancer with unique anti-inflammatory properties and demonstrated its therapeutic efficacy after oral administration in a murine IR model. However, most recent reports have focused on the use of oral IAP to treat intestinal inflammation-induced diseases such as metabolic syndrome (58) and hypertension (12). Oral IAP can be easily destroyed by acid pH (17). pH is increased by hydrotalcite as an intestinal alkalisation agent, and IAP activity improves; it has a certain remission effect on inflammation *in vivo* (15). Sung et al. (59) also found that CaCO_3_-based materials have potential for biomedical applications because of their safety and beneficial characteristics, such as pH sensitivity, CO_2_ gas generation, and antacid properties. Herein, to additionally incorporate antioxidant and anti-inflammatory functions, we found that inflammation was related to IR, so we speculated that the IAP enhancer can indirectly alleviate IR, and the deeper mechanism needs further study and discussion. We demonstrated that the extrusion–expansion mixture of food materials could increase the “milder” properties and achieve a slow-release effect similar to that of hydrotalcite. We also showed that the IAP enhancer could still reach the duodenum after it was exposed to gastric acid. IAP dephosphorylates LPS (60), ATP (11), and UDP (61). However, the effect of IAP on dephosphorylation has not been reported in the pH range (pH 5–8) in the small intestine of humans and other nucleoside triphosphates. In this study, IAP effectively dephosphorylated LPS and nucleoside triphosphate in alkaline pH; thus, IAP + IAP enhancer could be effectively used *in vitro*. Therefore, these results showed the efficacy and safety of IAP + IAP enhancer and demonstrated that the combination of IAP and IAP enhancer plays critical and complementary roles in providing multifaceted benefits of IAP dephosphorylation.

HFD is associated with an imbalance in the normal composition and number of microbes in the gut. Among them, gram-negative bacteria can produce intestinal LPS, and gram-positive bacteria can produce a large number of luminal ATP and other nucleotide triphosphates (62). Although we based our study on a previous work that highlighted the preventive role of oral IAP against HFD-induced IR, few studies have investigated the prevalence of the IAP enhancer in IR. Although oat and xylitol improved IR in previous studies, the low dose used in our study was insufficient to produce a significant effect in 4 weeks (63, 64). Nevertheless, our data indicated that IAP + IAP enhancer could be an effective oral supplement against intestinal inflammation, thus protecting the host from IR. IAP in the lumen moves from the proximal small intestine to the distal large intestine; then, it becomes excreted in the faeces (65). Therefore, the faecal IAP level can directly reflect the IAP level as an indicator of early diagnosis of IR and is defined as “real-time IAP analysis” (22). The results showed that all HFD groups had less faecal IAP activity than the LFD control groups; however, IAP activity significantly increased after the treatment, especially IAP + IAP enhancer. Thus, IAP deficiency was associated with IR. This result also verified that oral IAP supplements could be a prevention/intervention for IR. However, because of the high IAP level in the small intestine, oral IAP supplementation is insufficient to significantly increase the IAP level in the lumen. Therefore, other intervention approaches should include the upregulation of IAP in alkaline environments, which more likely increase IAP activities since such activities are higher in alkaline environments higher in alkaline environments than in acidic environments. The combined use of IAP + IAP enhancer showed that IAP not only supplemented but also promoted the *in situ* activation of IAP in the small intestine by increasing intestinal pH.

HFD administration led to an imbalance between pro-inflammatory and anti-inflammatory factors, and the anti-inflammatory effects of IAP + IAP enhancer were identified in this study. Previous studies demonstrated that IL-17 and IFNγ negatively affect the gut barrier function (30). IL-22 is reduced in the intestine of obese mice, and restoration of IL-22 decreases metabolic abnormality by targeting intestinal permeability and endotoxemia (66). Improvement in gut barrier function and increased IL-22 levels are associated with the increase in the gene expression of tight junctions, including claudin 1 and ZO-1 (67). Our data showed that IAP + IAP enhancer modulated the gut barrier function partly by controlling the levels of these cytokines and thus prevented intestinal inflammation. Tight-junction proteins, such as ZO-1, occludin, and claudin-1, are important factors regulating intestinal permeability and major barrier components. When pathogenic bacteria and viruses attack, they hijack TJ proteins to enter and infect cells and promote inflammatory factor production (68). Our results showed that HFD downregulated the gene expression of ZO-1, occludin, and claudin-1, resulting in gut structure deterioration. IAP + IAP enhancer showed significantly increased claudin-1, occludin, and ZO-1 caused by HFD administration. This effect was consistent with that observed by Sulaiman (69), who found that the loss of IAP expression is associated with the decreased expression of intestinal junctional proteins and impaired barrier function. We showed that intestinal permeability was controlled by IAP + IAP enhancer, which was inversely associated with ZO-1, occludin, and claudin-1 expression levels. These results further confirmed the conclusion that IAP + IAP enhancer might regulate intestinal inflammation by maintaining the barrier function *in vivo*.

**Figure 13:**
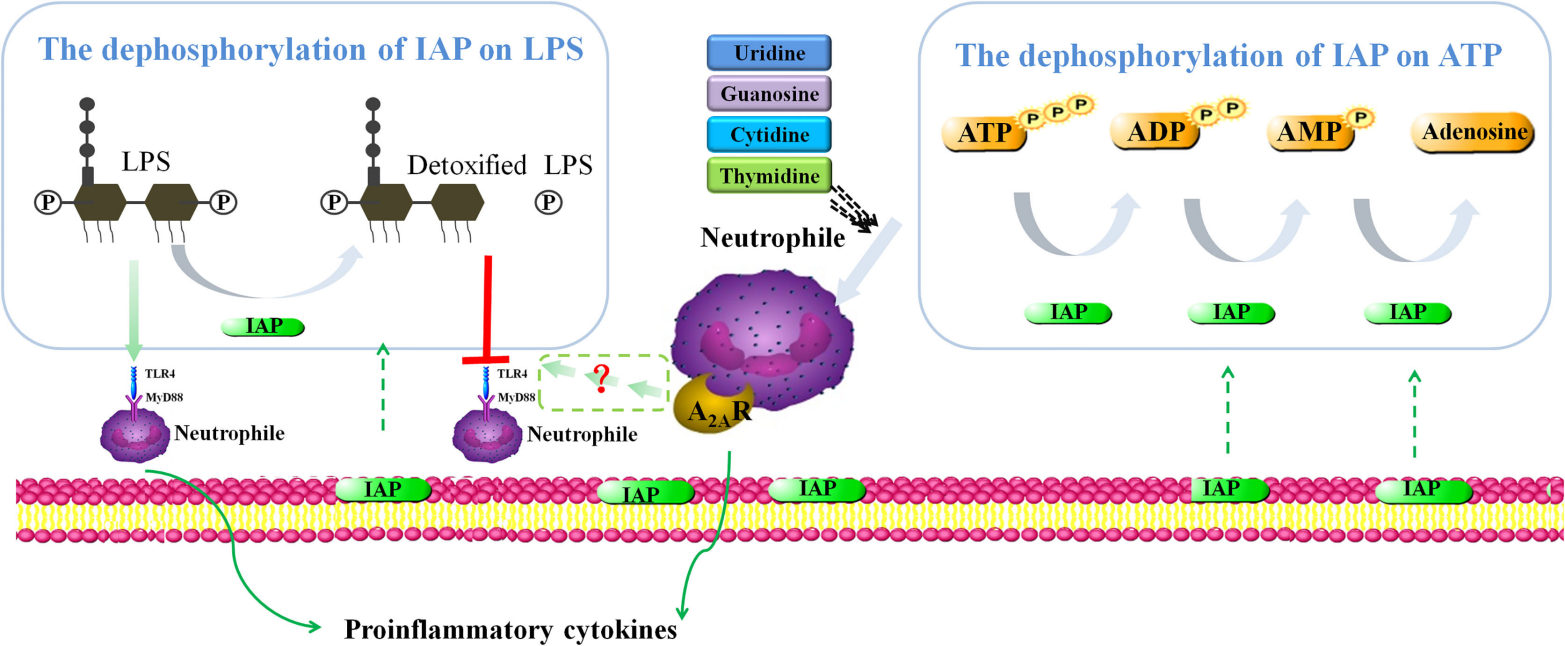
Proposed intestinal inflammation protective mechanism of IAP.

Two mechanisms could explain how IAP treatment alleviated intestinal inflammation *in vitro* (**Figure 13**), which led to IR. We mimicked LPS-induced intestinal inflammation that could demonstrate the IAP-mediated decrease in the LPS-induced cytokine production of TNF-α and IL-6. Interestingly, this study showed high levels of TNF-α/IL-6 production in unstimulated HT29 cells + leucocyte cocultures because neutrophils participate in the occurrence and maintenance of inflammation and release many inflammatory mediators. Activated neutrophils also release a large number of inflammatory mediators, such as cytokines, chemokines, and arachidonic acids, to promote inflammatory responses (70). The upregulated expression of NO derived from epithelial cells is related to the aggravation of IBD through iNOS (71). Moreover, the PGE_2_ level in inflammatory tissues increases compared with that in intact mucous membranes in the pathogenesis of inflammatory bowel disease (72). Furthermore, LPS could produce NO and PGE_2_, which were reduced after IAP treatment.

Under pathophysiological conditions, induced by inflammation or hypoxia, extracellular ATP release is enhanced, thereby accelerating inflammation and tissue injury (11). Adenosine produced *via* ATP hydrolysis has an anti-inflammatory property. Maria G Z et al. (73) found that guanosine is associated with its modulation of NF-κB nuclear translocation in DNBS-colitis rats. However, in this study, GTP dephosphorylation increased the level of inflammatory factors possibly because of the detection time point and different doses of GTP dephosphorylation. The relationship between TTP dephosphorylation and inflammation remains unclear and should be further discussed and verified. In addition, the hydrolysed products of other nucleotides of triphosphates have synergistic effects with adenosine. Sertac et al. (74) showed that uridine can activate the A_1_ receptor of adenosine; cytidine can activate A_1_ and A_2A_ receptors of adenosine and play an important role in cardiovascular and cerebrovascular diseases. Guanosine can increase extracellular adenosine levels, triggering its interaction to inhibit vascular smooth muscle cell proliferation (75). Although studies on the relationship between thymidine and adenosine have yet to be performed, our study suggested that thymidine might promote adenosine or adenosine receptors by producing a synergistic effect. The inhibitory effect of uridine, guanosine, cytidine, and thymidine on TNF-α and IL-6 was unstable. However, combined with adenosine, TNF-α and IL-6 production in the cell model, where adenosine had a synergistic effect with cytidine and thymidine, could be reduced effectively.

Understanding the underlying anti-inflammatory mechanisms of IAP is essential for clinical applications. However, the physiological and molecular mechanisms and related signalling pathways of IAP in intestinal inflammation *in vitro* have not been reported. In the present study, PI3kinase comprises a P110 catalytic subunit and a P85 regulatory subunit, which can be activated when P85 is activated (76); then, PI3K transmits signals to Akt, which induces a series of inflammatory responses. CXCL9 was downregulated in the IAP + LPS group upon binding to CXCR3 and CXCR3, resulting in Src phosphorylation and increasing the Src kinase activity; consequently, phosphatidylinositol 3 kinase (PI3K) activity increased, leading to an increase in Akt activity (37). LPAR1, SHC3, and EGF were downregulated in the adenosine group. LPAR1 binds to three G proteins (Gα12/13, Gα Q/11, and Gα I/O), thereby activating the Akt pathway (77). The interaction between SHC3 and GRB2-associated binding protein 1 (Gab1) activates the P85 subunit of PI3K and its downstream effector factor Akt, thus triggering the PI3K-Akt signalling pathway (52). The JAK-STAT signalling pathway is an immune-mediated inflammatory response triggered by dozens of cytokines (78). After the treatment of IAP + LPS and adenosine, the expression of IFN-stimulating genes (ISGs) was promoted by the JAK-STAT signalling pathway, and the IFNL1, IFNL2, and IFNL3 levels were significantly downregulated. The adenosine group also inhibited the non-receptor tyrosine kinase JAK family by inhibiting the dimerisation of THPO to MPL (54). CSF3 and CCL2 were downregulated in two groups. TRAF6 is inhibited by adenosine, while self-ubiquitinated TRAF6 binds to the TAK1 complex and activates the IKK complex; as a result, the NF-κB signalling pathway is activated (55). Numerous related proteins of NLRs are closely related to intestinal inflammation (79). In the IAP + LPS group, the NOD-like signalling pathway was inhibited by reducing HCK. However, the NOD2 content decreased after the adenosine treatment. HCK activates NLRP3 inflammasome by interacting with NLRP3 and promoting ASC oligomerisation (46); conversely, NOD2 consists of two N-terminal caspase activation and recruitment domains (CARDS), which initiate NF-κB by interacting with the serine and threonine kinase Rip2 (46). After IAP + LPS treatment, the expression of NF-κB signalling proteins and nuclear transcription factors, such as MEFV and S100A8, was downregulated in HT-29 cells. The MAPK signalling pathway mediates intracellular signalling related to various cellular activities by adenosine treatment (80). NF-κB and MAPK signalling pathways do not function alone (cross talk). For example, inhibitors of ERK1/2 and P38 signalling pathways can inhibit NF-κB activity and P65 nuclear transfer (81). NF-κB and MAPKS signalling pathways are the most common inflammatory signalling pathways.

Therefore, in this study, we found a new IR target, namely, IAP, which can dephosphorylate LPS and triphosphate nucleotide by supplementing and activating IAP in the intestinal tract; consequently, intestinal inflammation was controlled, and IR induced by intestinal inflammation was alleviated rather than control the gut microbes to alleviate IR and T2DM as other studies have reported.

In conclusion, IR mice exhibited signs of intestinal inflammation that were linked to unfavourable changes in IR, urine pH, faecal IAP, inflammatory markers, LPS in serum, and gut barrier function. This study revealed that oral IAP and IAP enhancer could interfere with IR by controlling intestinal inflammation. IAP effectively inhibited the inflammation caused by LPS, while adenosine, the dephosphorylation product of IAP, had anti-inflammatory effects. Thus, this study provided an important basis for the development and utilisation of IAP and for the further processing and application of food and pharmaceutical industry resources.

## Data availability statement

The datasets presented in this study can be found in online repositories. The name of the repository and accession number can be found below: NCBI Sequence Read Archive; accession PRJNA833108.

## Ethics statement

The studies involving human participants were reviewed and approved by Changchun Jiahe Surgical Hospital. The patients/participants provided their written informed consent to participate in this study. The animal study was reviewed and approved by Northeast Agricultural University Committee on Animal Resources.

## Author contributions

CG performed all cell studies and part of the animal experiment. MK performed the language checking and editing of the manuscript. WH performed analysed the animal experiment data. JL performed some physicochemical experiments. ND supervised the research. MH and MD analysed the data and wrote the manuscript. All authors read, revised (if needed), and approved the manuscript.

## Funding

Funding from this article was from Talent Introduction Program of Northeast Agricultural University, Study on the production and mechanism of antiinflammatory food B-HA/AP products.

## Acknowledgments

We are grateful to the family described in this manuscript for their willingness to participate and interest in this research. We thank Dr. Pei for reading and editing the manuscript. We acknowledge Pro Han and members of the Food college laboratory for help with physicochemical experiments. We thank Shaoxing Huihui Biotechnology Co. for help with cell experiments. We thank the Laboratory of Molecular Nutrition and Immunity for valuable comments and discussion.

## Conflict of interest

The authors declare that the research was conducted in the absence of any commercial or financial relationships that could be construed as a potential conflict of interest.

## Publisher’s note

All claims expressed in this article are solely those of the authors and do not necessarily represent those of their affiliated organizations, or those of the publisher, the editors and the reviewers. Any product that may be evaluated in this article, or claim that may be made by its manufacturer, is not guaranteed or endorsed by the publisher.
