# Supplementary material for: Intestinal alkaline phosphatase (IAP, IAP Enhancer) attenuates intestinal inflammation and alleviates insulin resistance
Source: Front Immunol. 2022 Jul 25;13:927272. doi: 10.3389/fimmu.2022.927272 (PMC9359302; doi:10.3389/fimmu.2022.927272)
Supplement: Supplementary file 1 [file DataSheet_1.doc]

**Online Data Supplement**

Tab. S1 Full list of IAP enhancer constituents

| Composition | Content(%) |
| --- | --- |
| Oatmeal | 34.5 |
| Shell powder | 26.75 |
| Aluminium hydroxide | 15.6 |
| Calcium carbonate | 10.92 |
| Magnesium carbonate | 6.17 |
| Xylitol | 6 |

Tab. S2 The reaction system for quantitative real-time PCR

| Reagent | Volume (μL) |
| --- | --- |
| cDNA (2 mmol/L) | 1.0 |
| Forward primer (4 μmol/L) | 1.0 |
| Reverse primer (4 μmol/L) | 1.0 |
| 2×SYBR® Green Supermix | 10.0 |
| ddH_2_O | Up to 20 |

Tab. S3 Quantitative real-time PCR primers

| Gene Name | Primer Sequence (5’to 3’) | |
| --- | --- | --- |
|  | Forward | Reverse |
| claudin-1 | 5′-TGGTAATTGGCATCCTGCTG-3′ | 5′-CAGCCATCCACATCTTCTGC-3′ |
| occludin | 5′-GTACCCACCAGTGACCAACA-3′ | 5′-GTTGCTGGAGCTTAGCCTGT-3′ |
| ZO-1 | 5′-CGAGGCATCATCCCAAATAAGAAC-3′ | 5′-TCCAGAAGTCTGCCCGATCAC-3′ |
| MTCP1 | 5’-TTCAGGTTCCCTTAGGTGAC-3’ | 5’-TGCTGTATCTGCCACAAGC-3’ |
| CXCL9 | 5’-CCAAGGGACTATCCACCTAC-3’ | 5’-CTGCTGAATCTGGGTTTAGAC-3’ |
| LY96 | 5’-TCTGAAGGGAGAGACTGTGAA-3’ | 5’-GTTGGTGTAGGATGACAAACTC-3’ |
| JAK2 | 5’-CTTGGGATGGCAGTGTTAG-3’ | 5’-ACCTTATTCGCTTCCTTGTC-3’ |

*
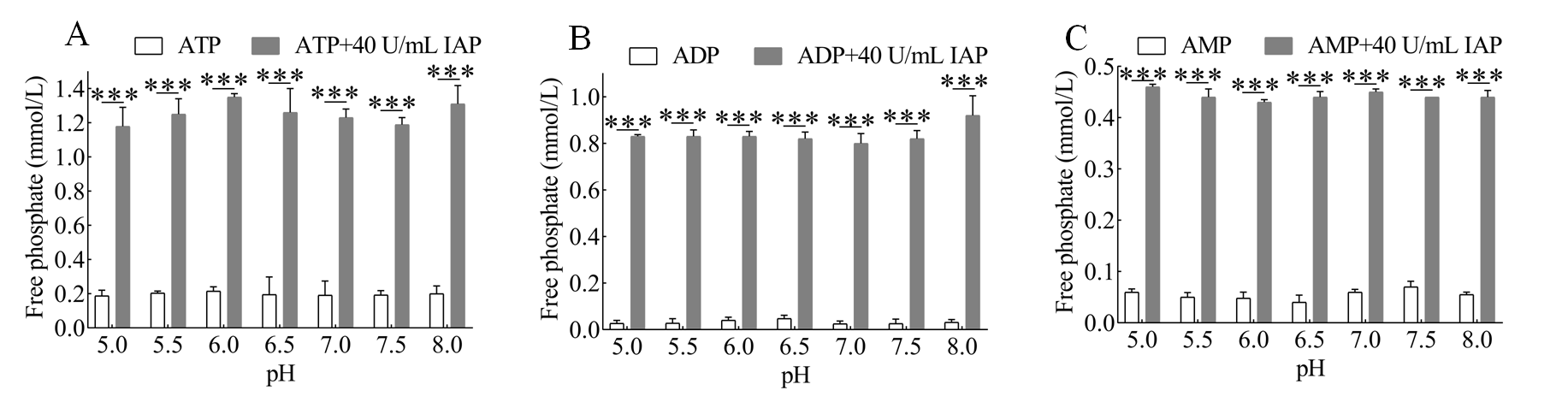
*

Fig. S1. The dephosphorisation of (A) ATP, (B) ADP, and (C) AMP by 40 U/ml IAP at different pH levels. Values are presented as means ± SD (n = 3/group). Two-tailed unpaired Student’s t test. ***P <0.001.

*
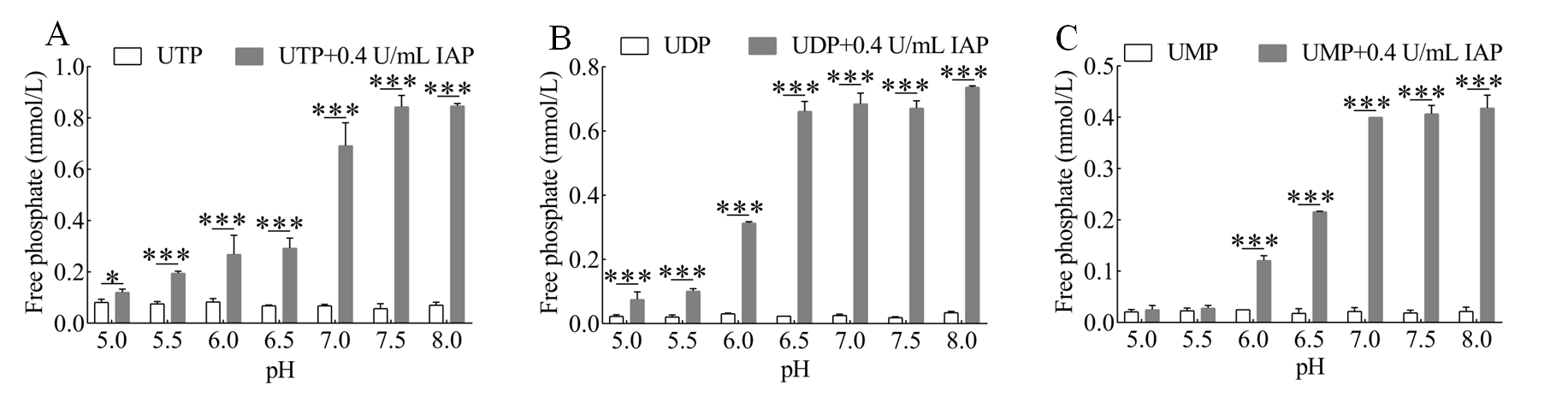
*

Fig. S2. The dephosphorisation of (A) UTP, (B) UDP, and (C) UMP by 0.4 U/ml IAP at different pH levels. Values are presented as means ± SD (n = 3/group). Two-tailed unpaired Student’s t test. *P <0.05;***P <0.001.

*
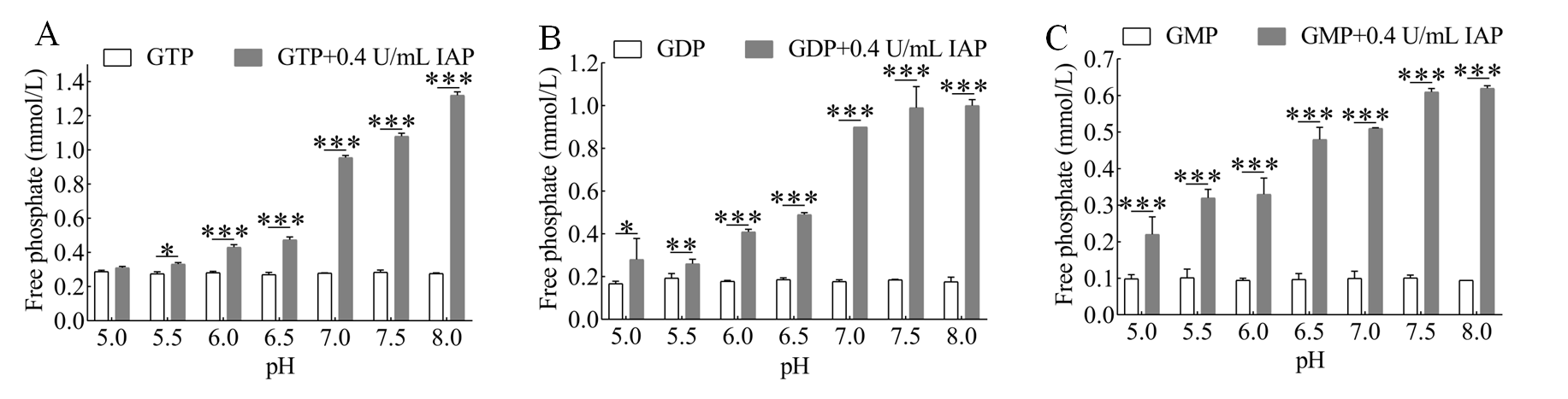
*

Fig. S3. The dephosphorisation of (A) GTP, (B) GDP, and (C) GMP by 0.4 U/ml IAP at different pH levels. Values are presented as means ± SD (n = 3/group). Two-tailed unpaired Student’s t test. *P<0.05; **P<0.01;***P<0.001.

*
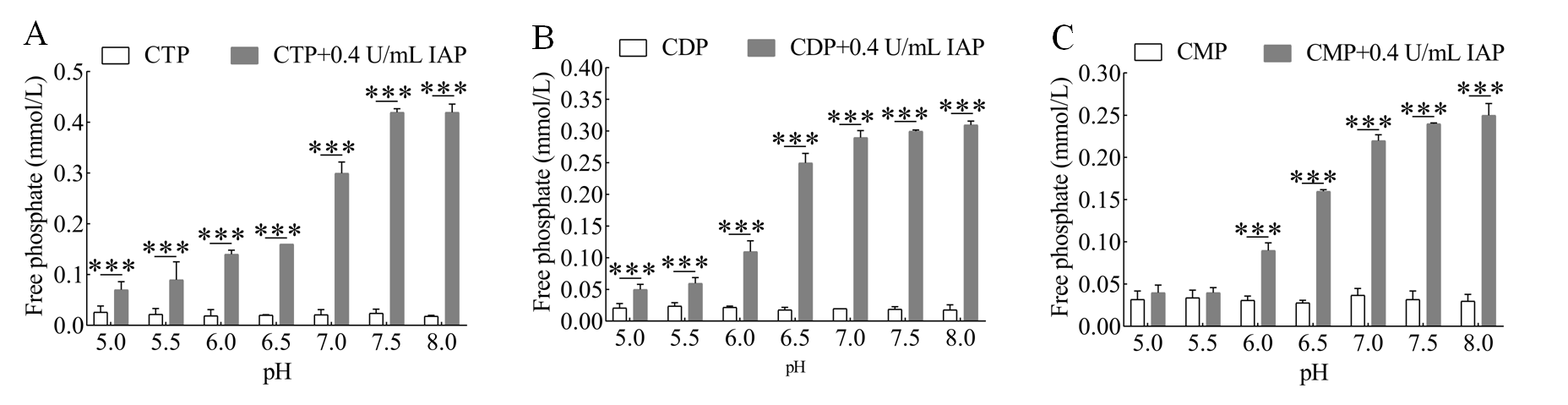
*

Fig. S4. The dephosphorisation of (A) CTP, (B) CDP, and (C) CMP by 0.4 U/ml IAP at different pH levels. Values are presented as means ± SD (n = 3/group). Two-tailed unpaired Student’s t test. ***P<0.001.

*
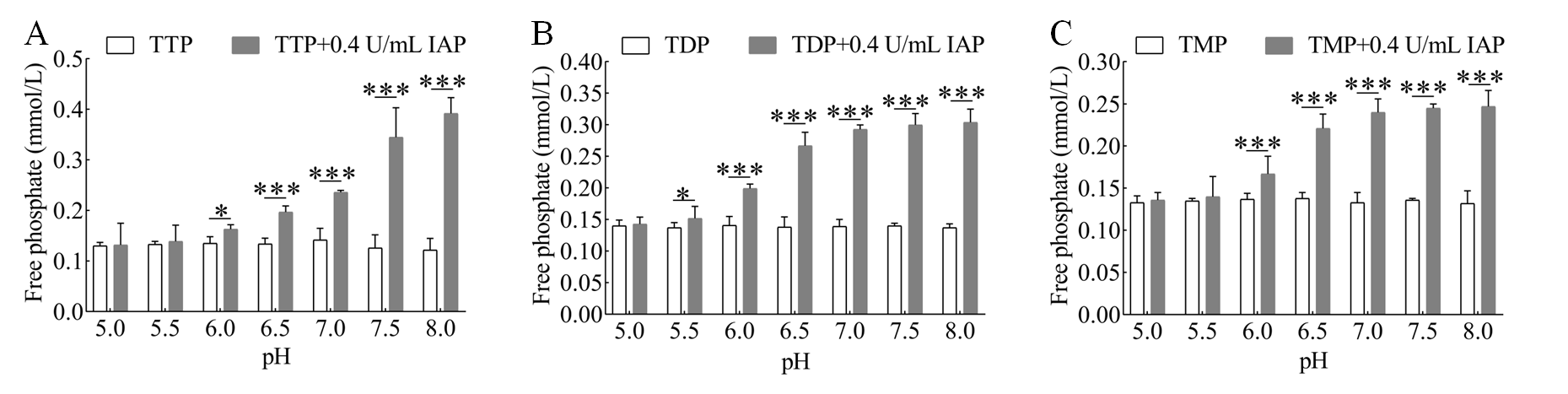
*

Fig. S5. The dephosphorisation of (A) TTP, (B) TDP, and (C) TMP by 0.4 U/ml IAP at different pH levels. Values are presented as means ± SD (n = 3/group). Two-tailed unpaired Student’s t test. *P<0.05; ***P<0.001.

*
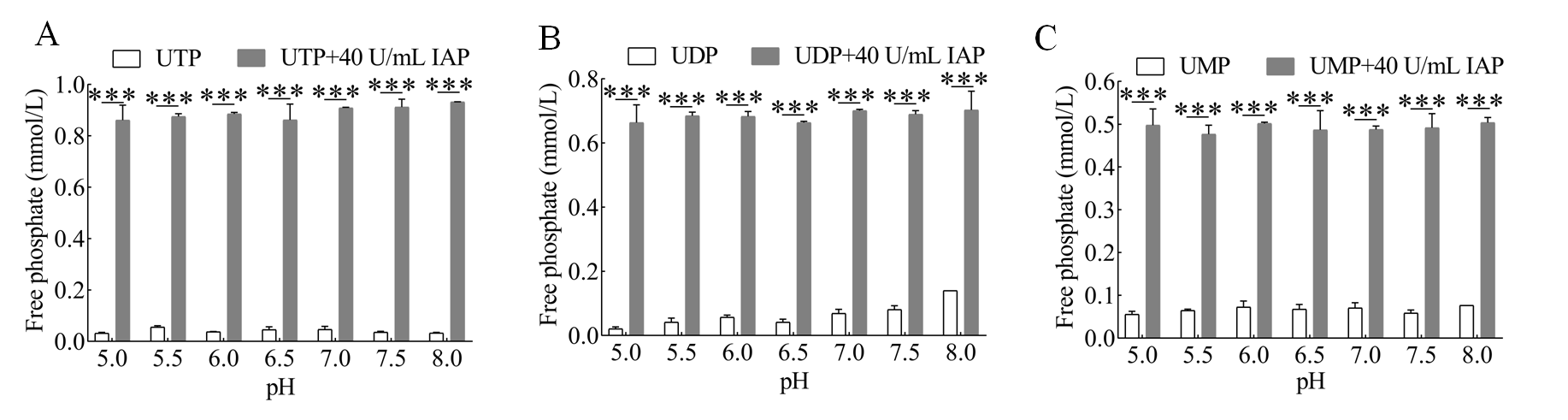
*

Fig. S6. The dephosphorisation of (A) UTP, (B) UDP, and (C) UMP by 40 U/ml IAP at different pH levels. Values are presented as means ± SD (n = 3/group). Two-tailed unpaired Student’s t test. ***P<0.001.

*
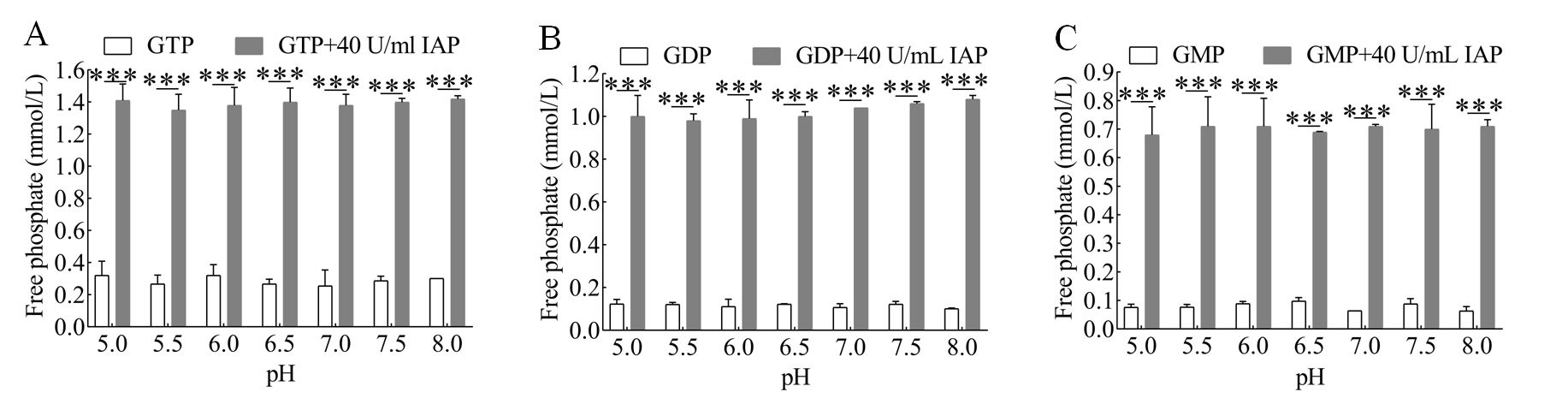
*

Fig. S7. The dephosphorisation of (A) GTP, (B) GDP, and (C) GMP by 40 U/ml IAP at different pH levels. Values are presented as means ± SD (n = 3/group). Two-tailed unpaired Student’s t test. ***P<0.001.

*
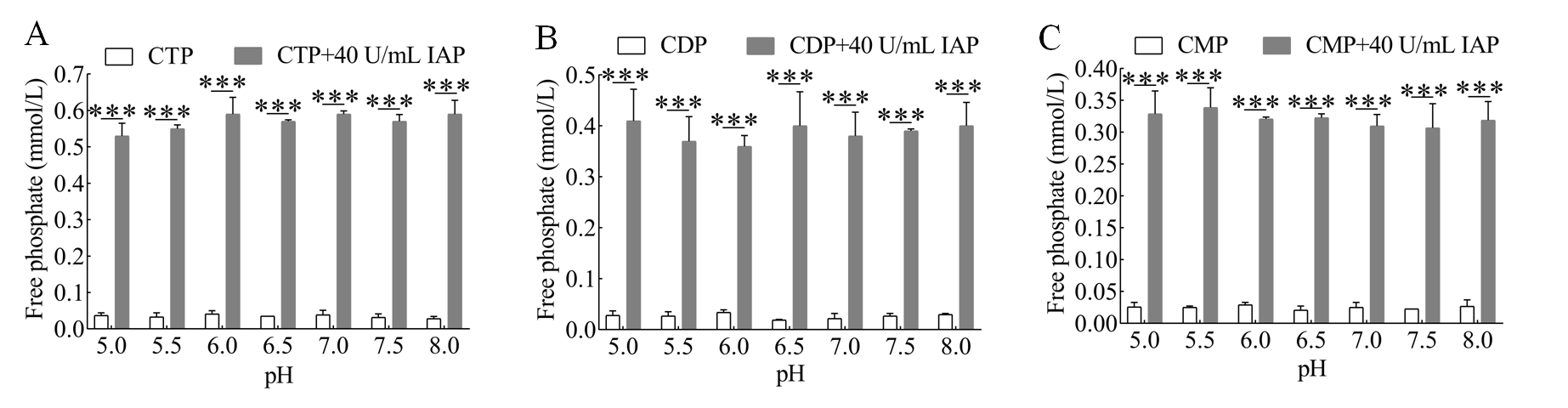
*

Fig. S8. The dephosphorisation of (A) CTP, (B) CDP, and (C) CMP by 40 U/ml IAP at different pH levels. Values are presented as means ± SD (n = 3/group). Two-tailed unpaired Student’s t test. ***P<0.001.

*
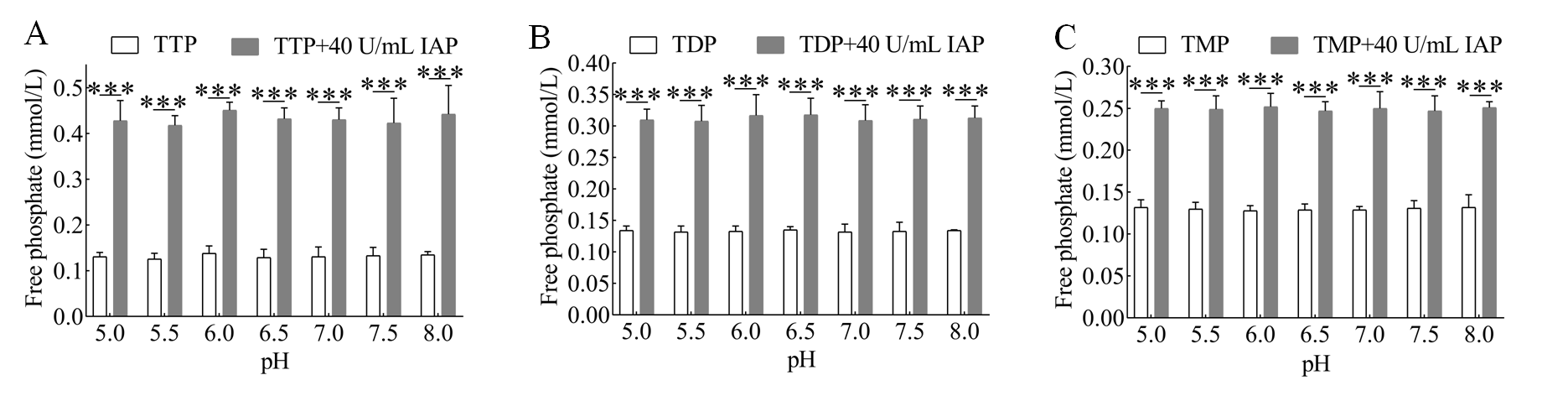
*

Fig. S9. The dephosphorisation of (A) TTP, (B) TDP, and (C) TMP by 40 U/ml IAP at different pH levels. Values are presented as means ± SD (n = 3/group). Two-tailed unpaired Student’s t test. ***P<0.001.


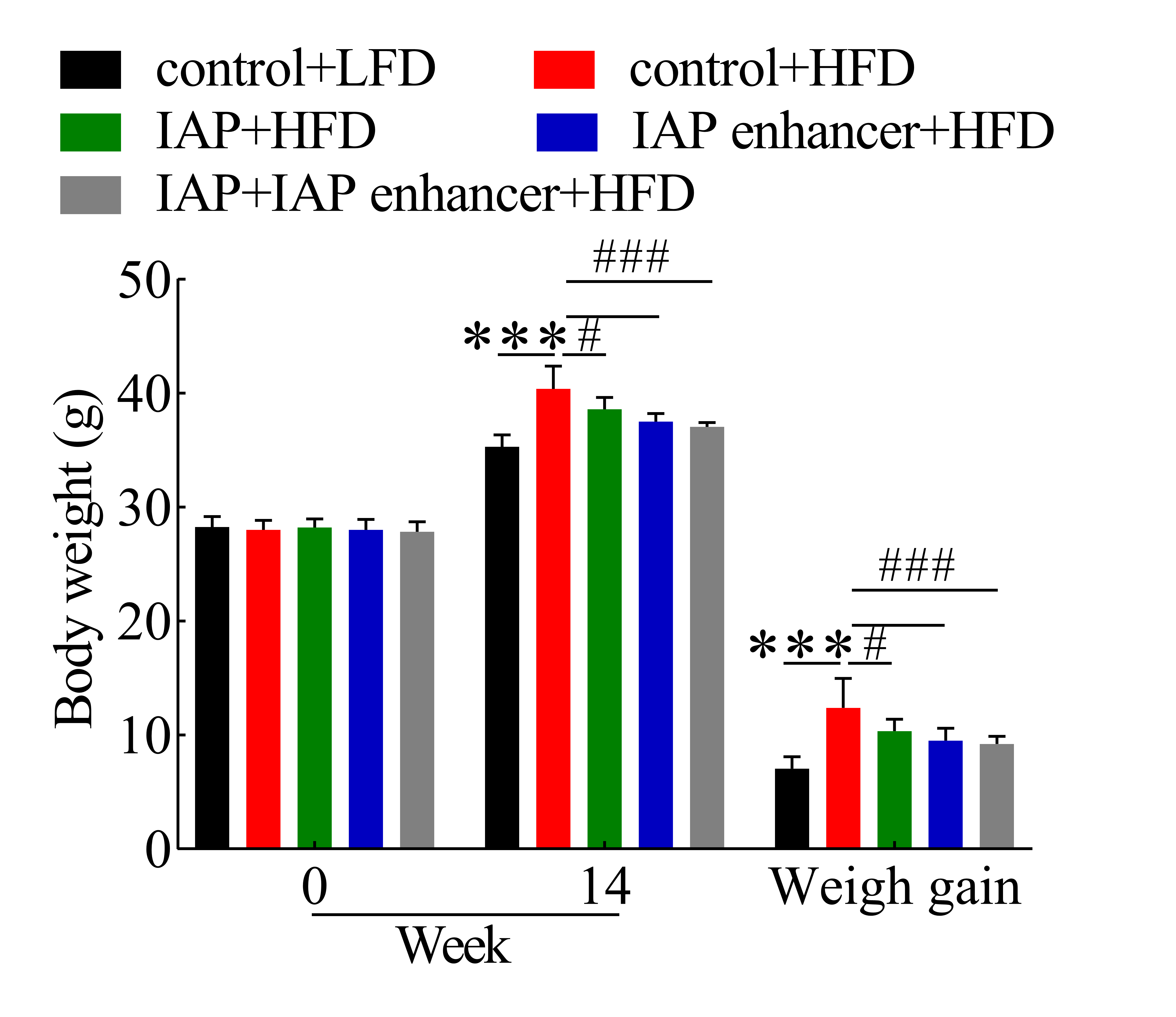


Fig. S10. The changes of body weight after intervention. Values are presented as means ± SD. Two-tailed unpaired Student’s t test. HF control groups compared with the LF control groups, ***P<0.001; HF treatment groups compared with the HF control groups, ^#^P<0.05, ^###^P<0.001.


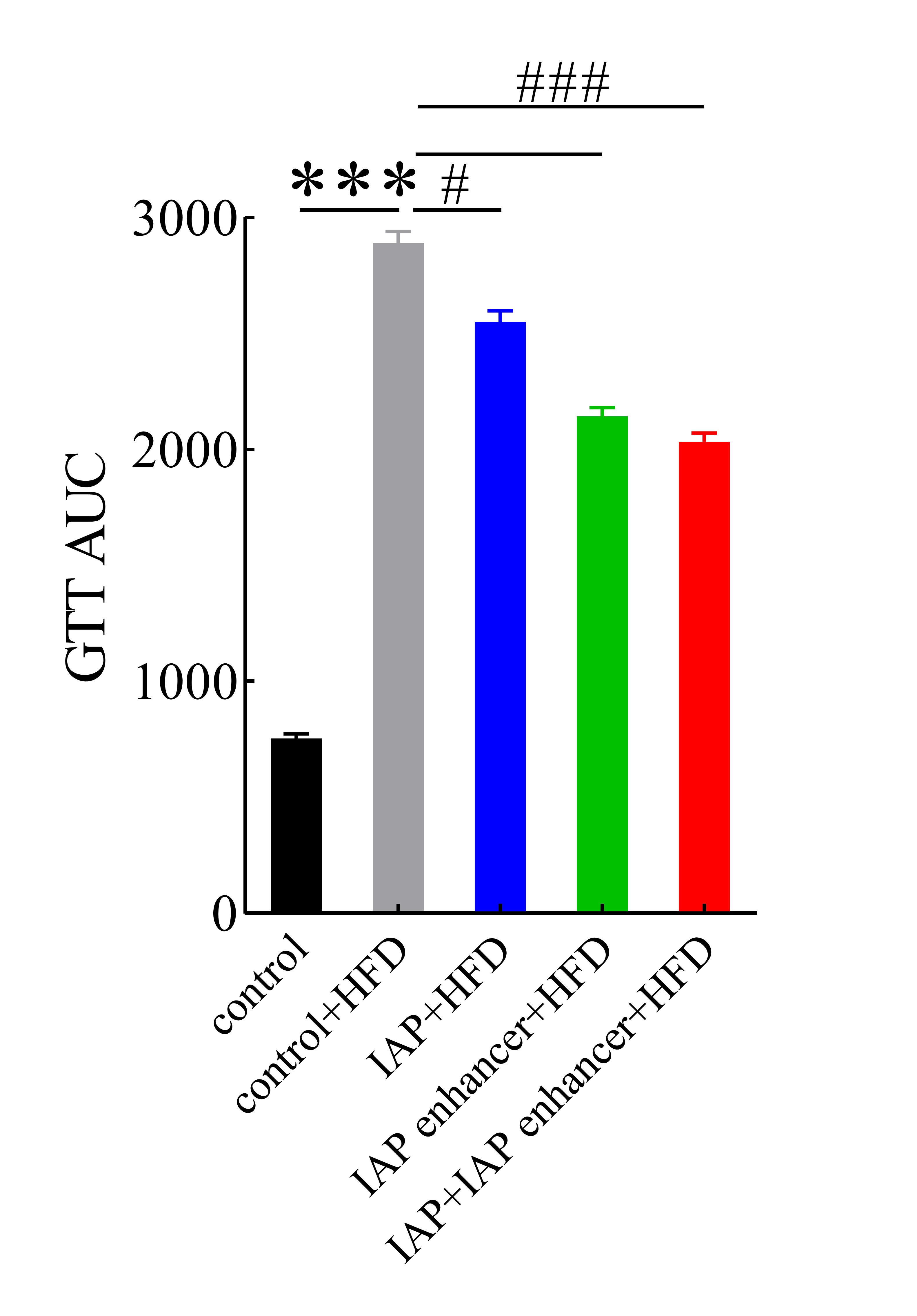


Fig. S11. The changes of AUC of GTT after intervention. Values are presented as means ± SD. Two-tailed unpaired Student’s t test. HF control groups compared with the LF control groups, ***P<0.001; HF treatment groups compared with the HF control groups, ^#^P<0.05, ^###^P<0.001.


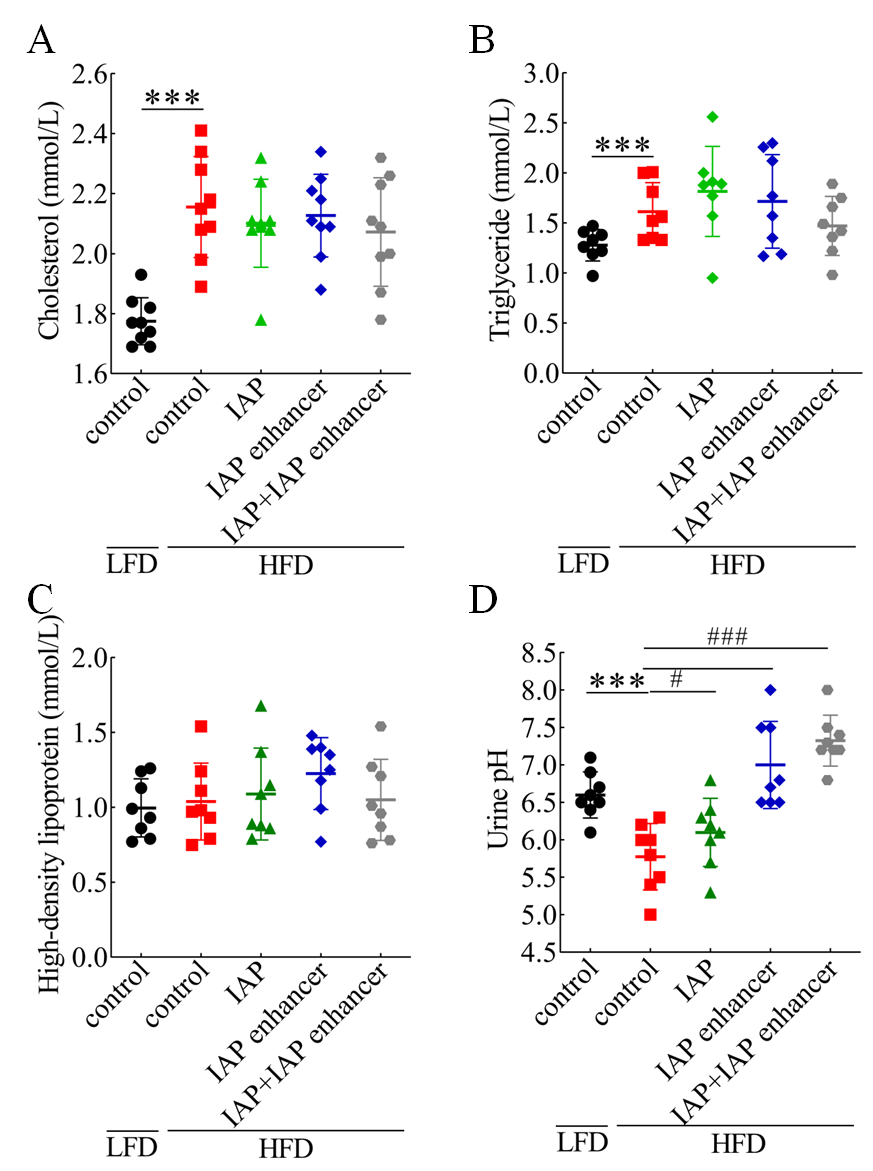


Fig. S12. The changes of lipid profile and urine pH after intervention. (A) Cholesterol, (B) Triglyceride, (C) High-density lipoprotein, (D) Urine pH. Values are presented as means ± SD. Two-tailed unpaired Student’s t test. HF control groups compared with the LF control groups, ***P<0.001; HF treatment groups compared with the HF control groups, ^#^P<0.05, ^###^P<0.001.

*
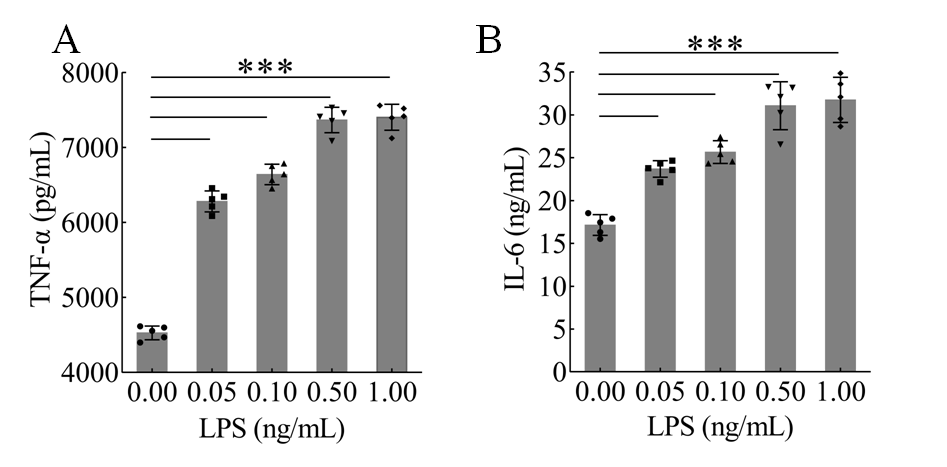
*

Fig. S13. LPS stimulates on the secretion of (A) TNF-α and (B) IL-6 release in co-cultured HT-29 cells + freshly extracted human leucocytes. Values are presented as means ± SD (n = 5 wells/group). Analysis of variance with Tukey. ***P<0.001.

*
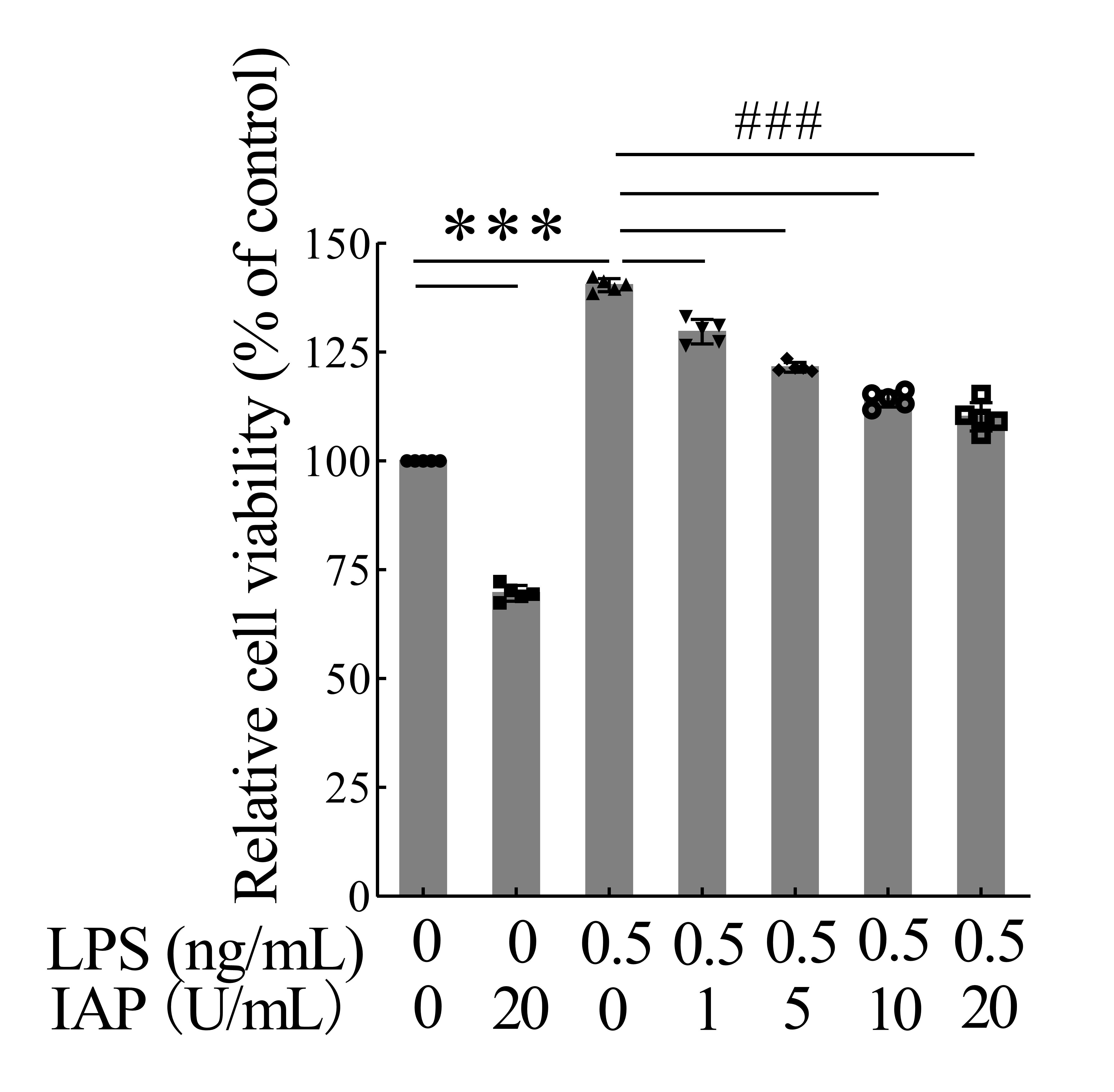
*

Fig. S14. Effect of different IAP activities on LPS-induced cell vitality in co-cultured HT-29 cells + freshly extracted human leucocytes. Values are presented as means ± SD (n = 5 wells/group). Analysis of variance with Tukey. Compared with the control, ***P<0.001, compared with the LPS, ^###^P<0.001.

*
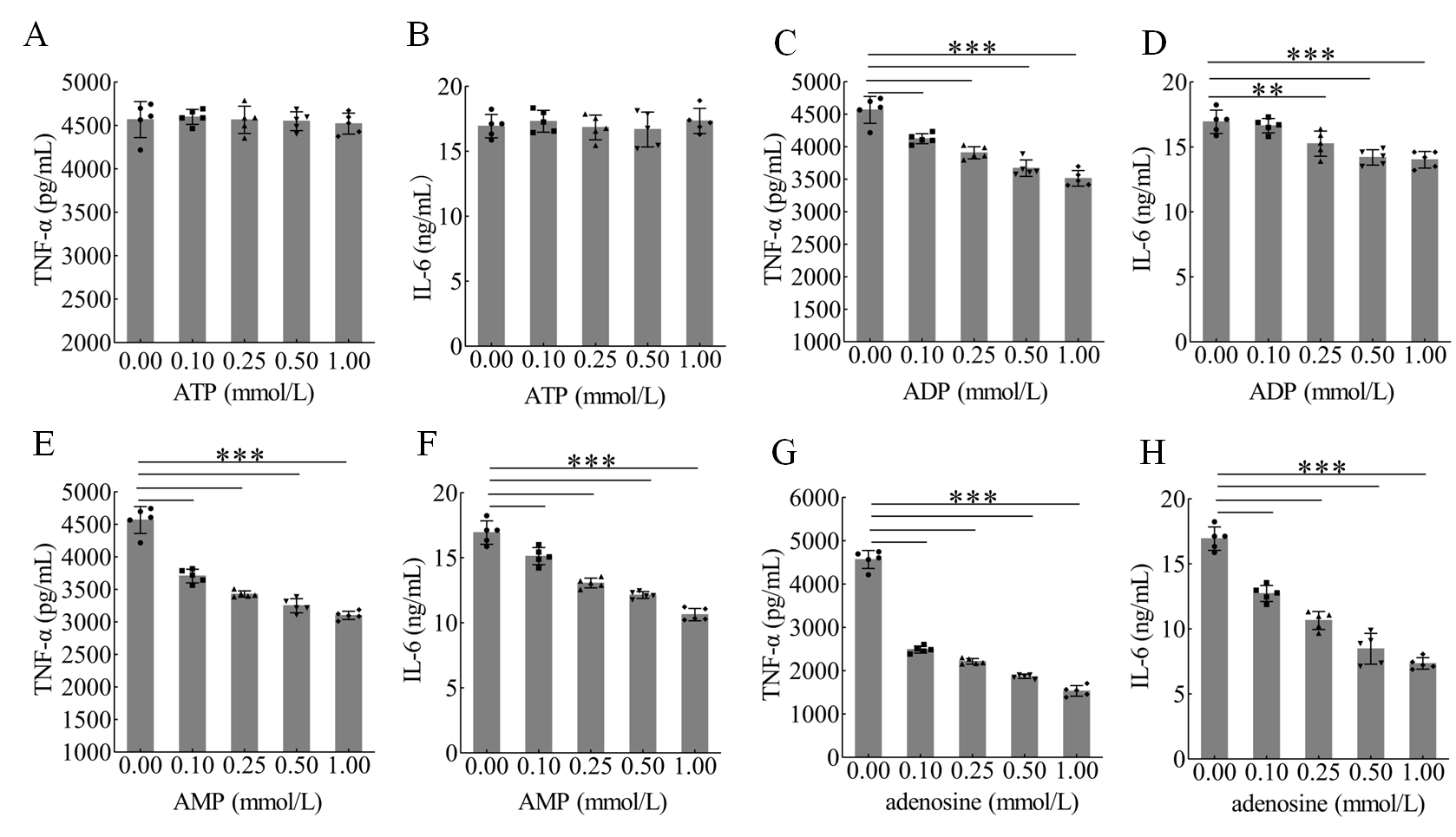
*

Fig. S15. Effect of (A B) ATP, (C D) ADP, (E F) AMP and (G H) adenosine on the secretion of TNF-α and IL-6 in co-cultured HT-29 cells + freshly extracted human leucocytes. Values are presented as means ± SD (n = 5 wells/group). Analysis of variance with Tukey. **P<0.01; ***P<0.001.


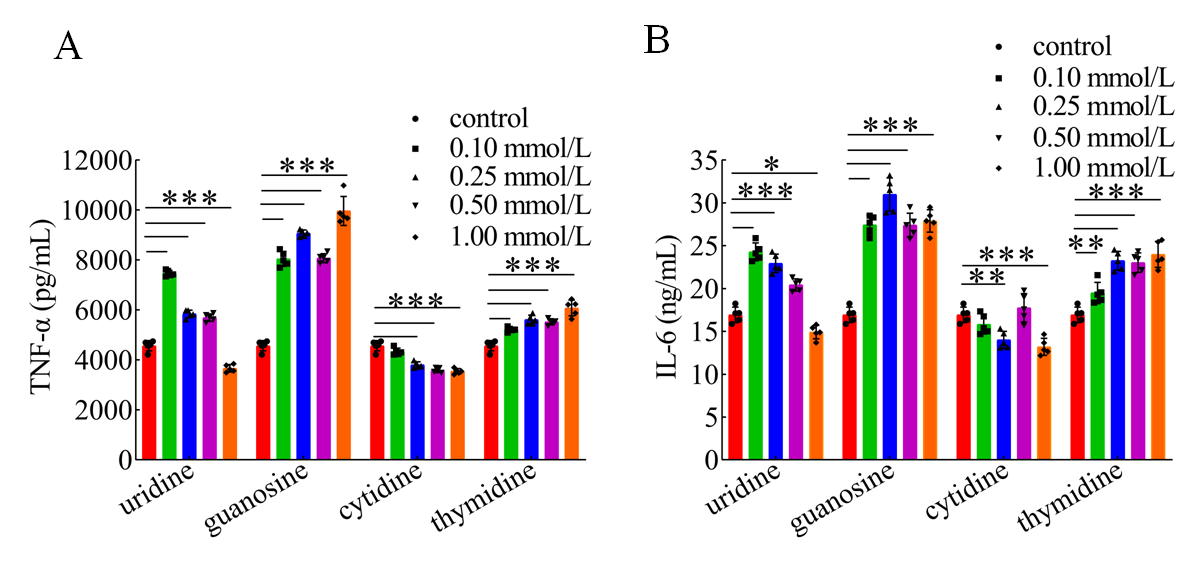


Fig. S16. Effect of uridine, guanosine, cytidine, and thymidine on the secretion of (A) TNF-α and (B) IL-6 in co-cultured HT-29 cells + freshly extracted human leucocytes. Values are presented as means ± SD (n = 5 wells/group). Analysis of variance with Tukey. *P<0.05; **P<0.01; ***P<0.001.

*
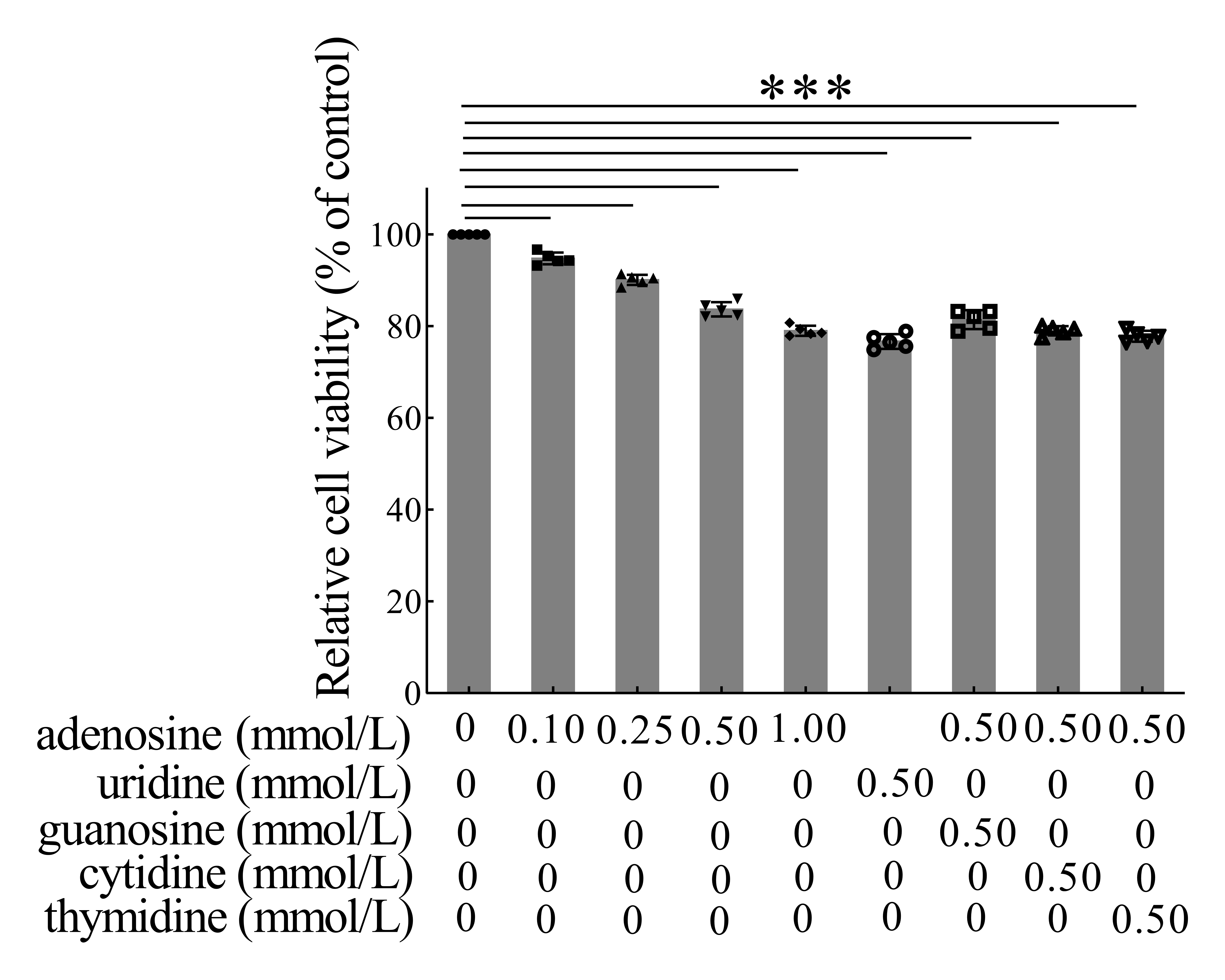
*

Fig. S17. Effect of adenosine and other nucleosides on cell vitality in co-cultured HT-29 cells + freshly extracted human leucocytes. Values are presented as means ± SD (n = 5 wells/group). Analysis of variance with Tukey. Compared with the control. ***P<0.001.

*
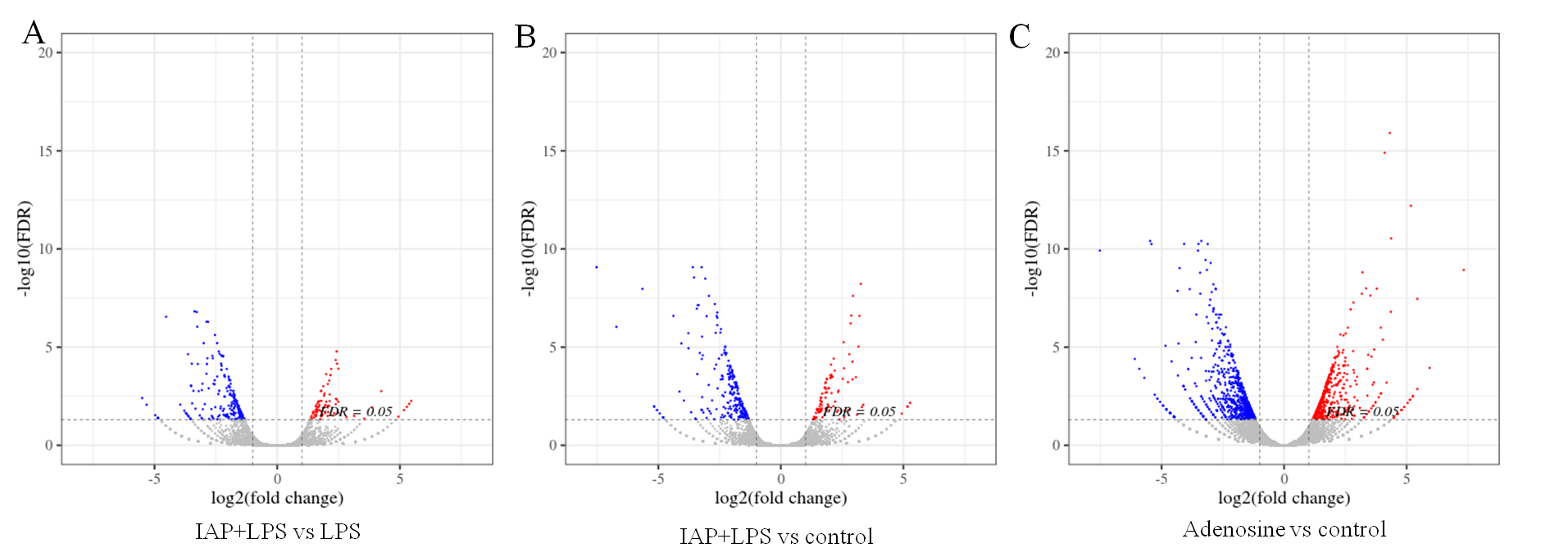
*

Fig. S18. Analysis of gene differential level. The volcano diagram of differentially expressed genes. (A) IAP+LPS vs. LPS, (B) IAP+LPS vs. control, (C) Adenosine vs. control.


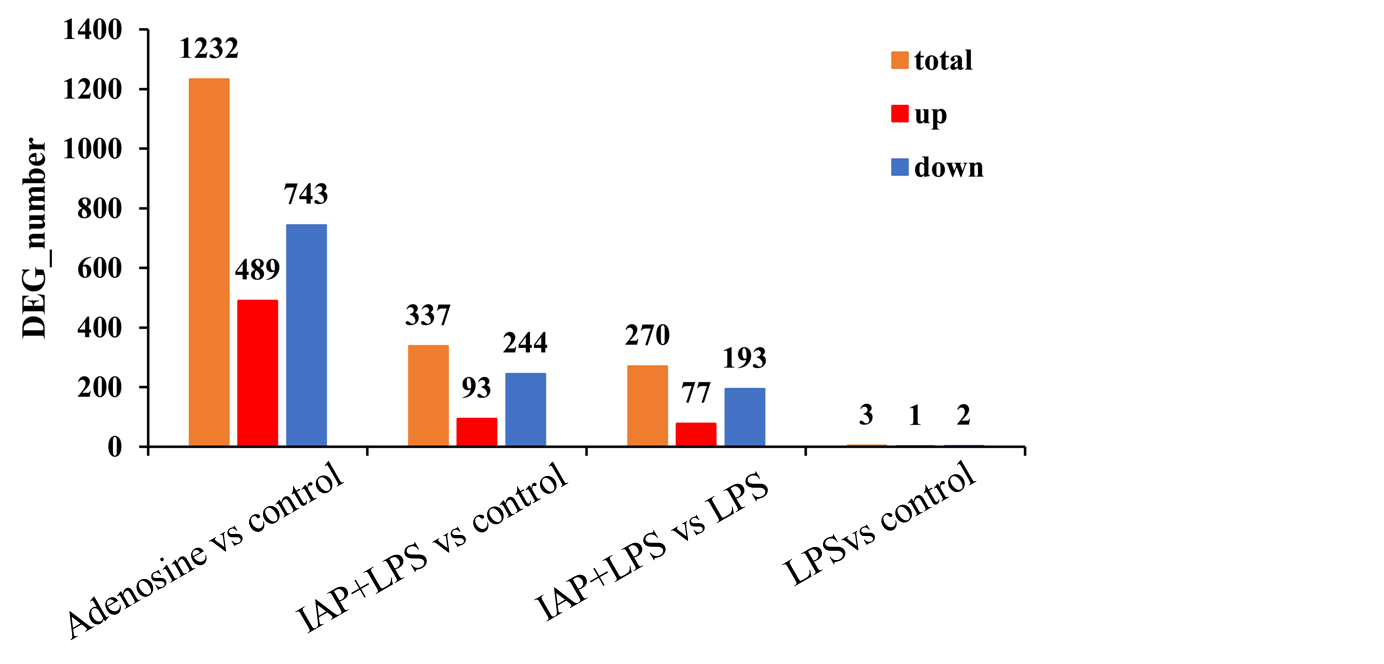


Fig. S19. Histogram of the number of [DEGs](javascript:;).

*
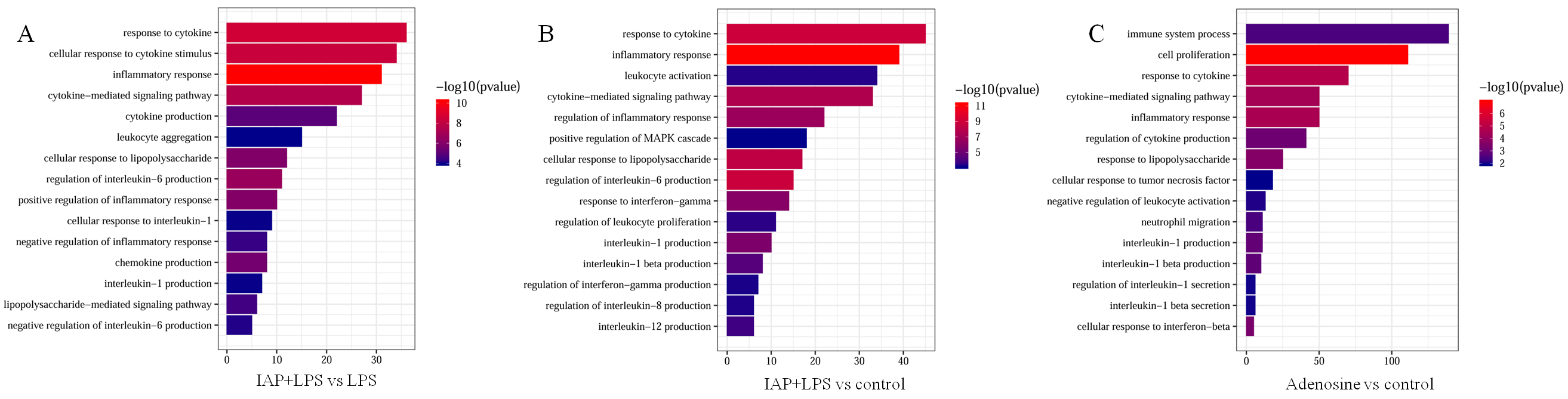
*

Fig. S20. Biological process related to inflammation GO enrichment. (A) IAP+LPS vs. LPS, (B) IAP+LPS vs. control, and (C) adenosine vs. control.

*
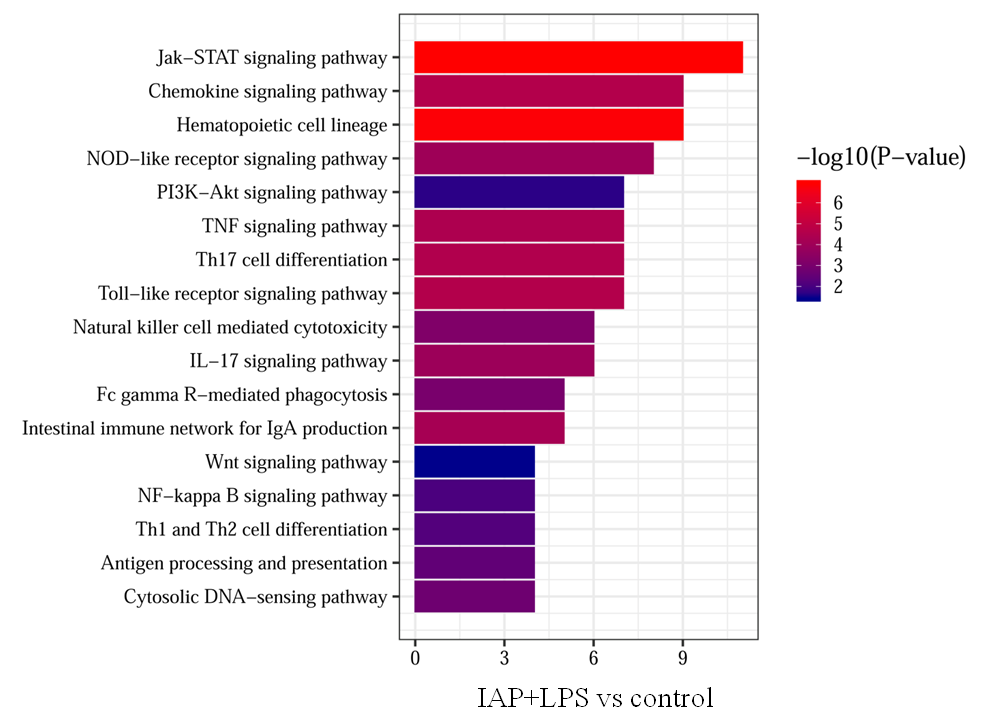
*

Fig. S21. Signalling pathways related to immune system and signal transduction KEGG enrichment of IAP+LPS vs. control.

*
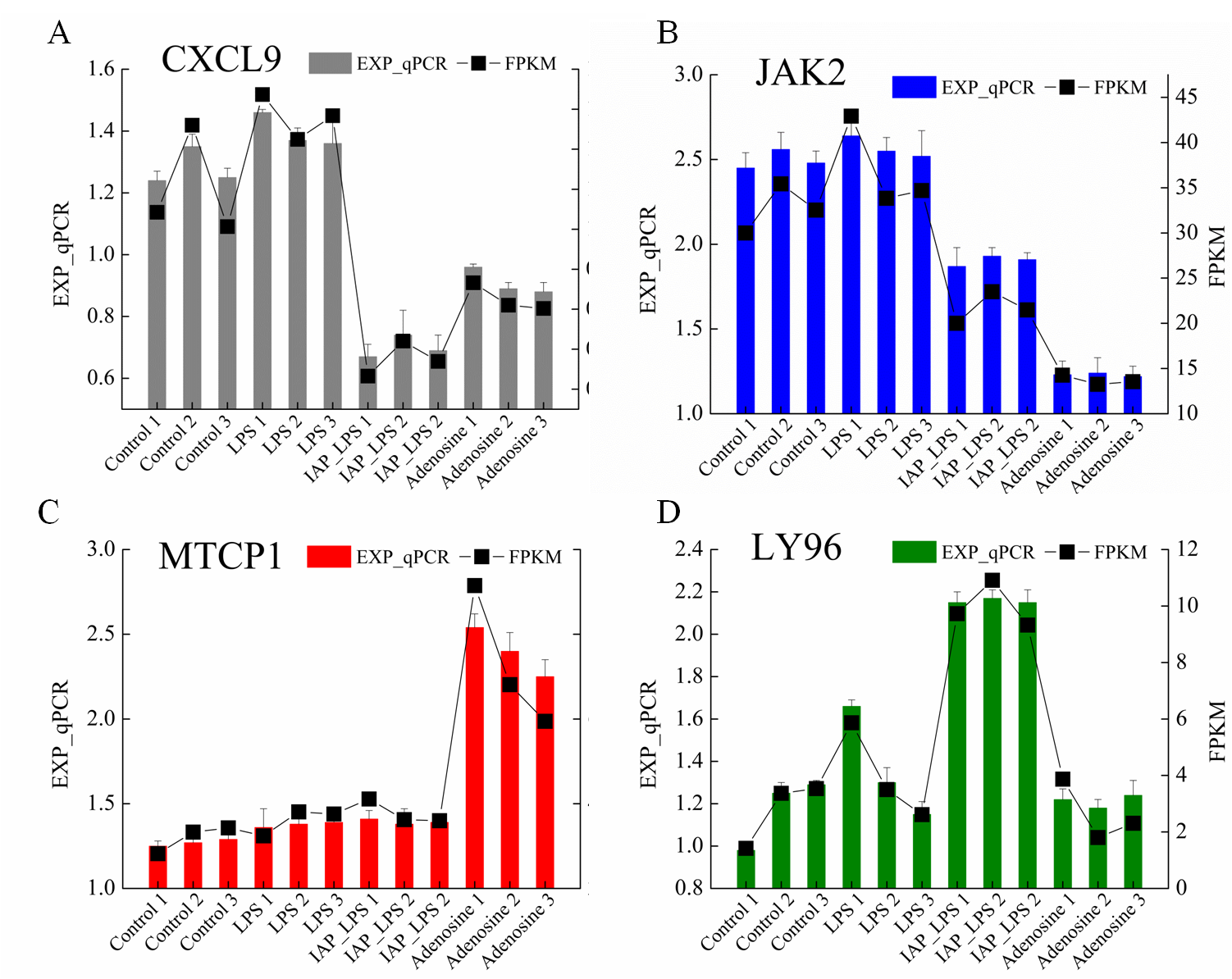
*

Fig. S22. The qPCR verification results of differentially expressed genes.
